# Cholesterol 25‐Hydroxylase inhibits SARS‐CoV‐2 and other coronaviruses by depleting membrane cholesterol

**DOI:** 10.15252/embj.2020106057

**Published:** 2020-10-05

**Authors:** Shaobo Wang, Wanyu Li, Hui Hui, Shashi Kant Tiwari, Qiong Zhang, Ben A Croker, Stephen Rawlings, Davey Smith, Aaron F Carlin, Tariq M Rana

**Affiliations:** ^1^ Division of Genetics Department of Pediatrics Institute for Genomic Medicine Program in Immunology University of California San Diego La Jolla CA USA; ^2^ Department of Biology University of California San Diego La Jolla CA USA; ^3^ Bioinformatics Program University of California San Diego La Jolla CA USA; ^4^ Division of Allergy, Immunology, and Rheumatology Department of Pediatrics University of California San Diego La Jolla CA USA; ^5^ Division of Infectious Diseases and Global Public Health Department of Medicine University of California San Diego La Jolla CA USA

**Keywords:** cholesterol 25‐hydroxylase, COVID‐19 treatment, innate immunity, restriction factor of coronaviruses, viral fusion, Membrane & Intracellular Transport, Microbiology, Virology & Host Pathogen Interaction

## Abstract

Coronavirus disease 2019 (COVID‐19) is caused by SARS‐CoV‐2 and has spread across the globe. SARS‐CoV‐2 is a highly infectious virus with no vaccine or antiviral therapy available to control the pandemic; therefore, it is crucial to understand the mechanisms of viral pathogenesis and the host immune responses to SARS‐CoV‐2. SARS‐CoV‐2 is a new member of the betacoronavirus genus like other closely related viruses including SARS‐CoV and Middle East respiratory syndrome coronavirus (MERS‐CoV). Both SARS‐CoV and MERS‐CoV have caused serious outbreaks and epidemics in the past eighteen years. Here, we report that one of the interferon‐stimulated genes (ISGs), cholesterol 25‐hydroxylase (*CH25H*), is induced by SARS‐CoV‐2 infection *in vitro* and in COVID‐19‐infected patients. CH25H converts cholesterol to 25‐hydrocholesterol (25HC) and 25HC shows broad anti‐coronavirus activity by blocking membrane fusion. Furthermore, 25HC inhibits USA‐WA1/2020 SARS‐CoV‐2 infection in lung epithelial cells and viral entry in human lung organoids. Mechanistically, 25HC inhibits viral membrane fusion by activating the ER‐localized acyl‐CoA:cholesterol acyltransferase (ACAT) which leads to the depletion of accessible cholesterol from the plasma membrane. Altogether, our results shed light on a potentially broad antiviral mechanism by 25HC through depleting accessible cholesterol on the plasma membrane to suppress virus–cell fusion. Since 25HC is a natural product with no known toxicity at effective concentrations, it provides a potential therapeutic candidate for COVID‐19 and emerging viral diseases in the future.

## Graphical abstract

Interferon‐induced cholesterol hydroxylation, and subsequent activation of the ER‐localized ACAT enzyme that depletes accessible cholesterol, represents a new host defence mechanism restricting membrane fusion and entry of severely pathogenic coronaviruses.

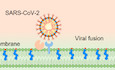

## Introduction

Coronavirus disease 2019 (COVID‐19) is a transmissible respiratory disease caused by a novel severe acute respiratory syndrome coronavirus SARS‐CoV‐2. Since its emergence in December 2019, COVID‐19 has rapidly spread worldwide spanning 216 countries (Sun *et al*, 2020). As of September 7^th^, there are 27,236,916 confirmed cases and 891,031 deaths (WHO COVID Pandemic). There is currently no approved vaccine for this disease, and there is an urgent need for developing therapeutic strategies. The etiologic agent of COVID‐19, SARS‐CoV‐2, is an enveloped positive‐sense single‐stranded RNA virus. SARS‐CoV‐2 is a new member of the betacoronavirus genus and other closely related viruses include SARS‐CoV and Middle East respiratory syndrome coronavirus (MERS‐CoV). Both SARS‐CoV and MERS‐CoV caused serious outbreaks and epidemics in the past eighteen years (Cui *et al*, 2019). SARS‐CoV‐2 uses angiotensin‐converting enzyme 2 (ACE2) as a host cell receptor, and transmembrane protease, serine 2 (TMPRSS2) to cleave its spike (S) protein for entry into the cell (Hoffmann *et al*, 2020b).

Interferons (IFNs) are induced upon coronavirus infections (Park & Iwasaki, 2020). IFNs are signaling molecules of the innate immune system that confer a first line of antiviral defense (Liu *et al*, 2013). Upon sensing of molecular patterns associated with viral infections, IFN pathways become activated, engendering further activation of hundreds of interferon‐stimulated genes (ISGs) that counteract many steps in the virus life cycle (Liu *et al*, 2013). Human type I IFNs including IFN‐α and IFN‐β function through the ubiquitously expressed type I interferon receptor (IFNAR), while type III IFNs, IFN‐λ, bind to the epithelia‐restricted type III interferon receptor (IFNLR; de Weerd & Nguyen, 2012). *In vitro* and clinical studies have shown that SARS‐CoV‐2 is sensitive to type I IFNs and that type I IFN treatment could be a promising therapeutic strategy for COVID‐19 (Mantlo *et al*, 2020; preprint: Meng *et al*, 2020; preprint: Zhou *et al*, 2020).

Cholesterol‐25‐hydroxylase (CH25H) is one of the ISGs (Schneider *et al*, 2014), which encodes an enzyme that synthesizes the oxysterol 25‐hydroxycholesterol (25HC) from cholesterol (Lund *et al*, 1998). 25HC has been shown to have broad antiviral activity by inhibiting the host cell entry of human immunodeficiency virus (HIV), vesicular stomatitis virus (VSV), Zika virus (ZIKV), Ebola virus (EBOV), Nipah virus (NiV), Russian spring‐summer encephalitis virus (RSSEV), porcine viruses (PEDV, PEGV, porcine intestine coronavirus), reovirus, and norovirus (Liu *et al*, 2013; Li *et al*, 2017). For ZIKV, treatment with 25HC protected mice and rhesus monkeys from ZIKV infection (Li *et al*, 2017). For the mechanism of entry inhibition, there is evidence that 25HC could modify plasma membrane composition (Liu *et al*, 2013). However, the pathways mediating 25HC‐induced modification of the plasma membrane that inhibits virus entry are unclear.

In this paper, we report the antiviral activity of CH25H against SARS‐CoV‐2 entry into human lung epithelial cells and show that 25HC broadly inhibits infection by human coronaviruses including SARS‐CoV and MERS‐CoV. By addressing the mechanism of 25HC function, we provide evidence that 25HC induced depletion of cholesterol on the plasma membrane through activating acyl‐CoA:cholesterol acyltransferase (ACAT), thereby restricting virus fusion at the cell surface. We further confirmed the inhibitory effect of 25HC on authentic SARS‐CoV‐2 infection of lung epithelial cells and viral entry in human lung organoids. These results shed light on one of the mechanisms by which interferon signaling and type I interferon therapy in COVID‐19‐infected patients counteract SARS‐CoV‐2 at the molecular level, with implications for cholesterol homeostasis in broad‐spectrum antiviral immunity.

## Results

### *CH25H* is induced by SARS‐CoV‐2 infection and restricts viral entry

**Figure 1:**
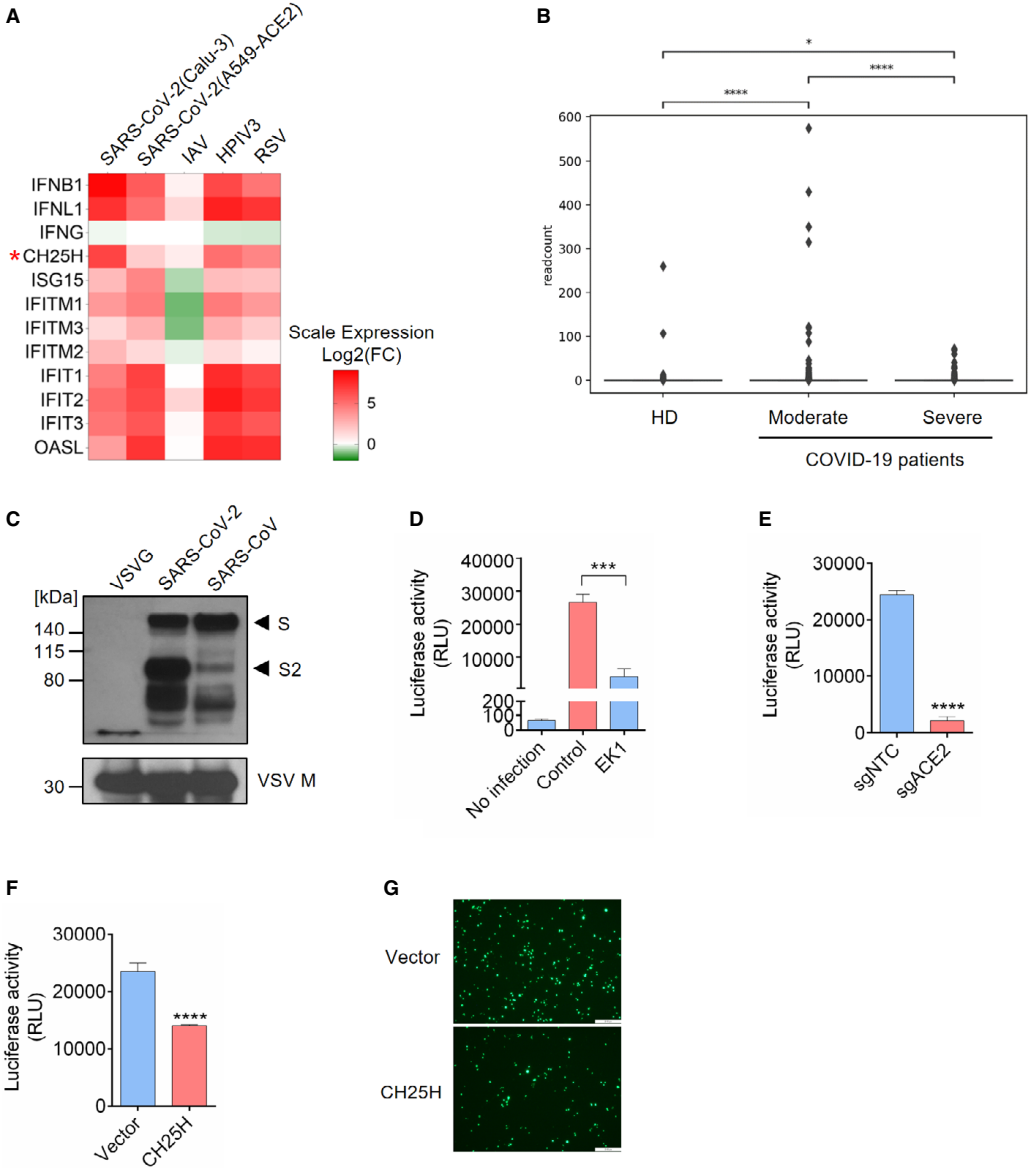
*Cholesterol 25‐hydroxylase (CH25H)* is induced by SARS‐CoV‐2 and restricts viral infection AIFNs and ISGs were induced by SARS‐CoV-2 infection in lung epithelial cell lines: Calu‐3 and A549‐ACE2 were infected with SARS‐CoV-2 at MOI = 2 for 24 h; A549 was challenged with IAV at MOI = 5 for 9 h; A549 was infected with HPIV3 and RSV at MOI = 2 for 24 h (Blanco‐Melo *et al*, 2020). Color key represents log2(fold change) relative to uninfected control cells. *CH25H* was highlighted by red asterisk.BExpression of *CH25H* in heathy donors and COVID‐19-infected patients. The box plot shows the expression of *CH25H* in macrophages of bronchoalveolar lavage fluids from four healthy donors, three moderate COVID‐19-infected patients and six severe COVID‐19-infected patients by scRNA‐seq analysis (Liao *et al*, 2020). *P < 0.05, ****P < 0.0001, by Student's *t*‐test.C–ECharacterization of SARS‐CoV-2 pseudovirus. (C) SARS‐CoV-2, SARS‐CoV, and VSV pseudovirus (VSV‐G) were concentrated by ultracentrifuge. Incorporation of spike protein on viral membrane was analyzed by Western blot using an antibody recognizing S2 subunit of SARS‐CoV and SARS‐CoV-2. VSV Matrix protein served as a loading control for viral particles. Black arrows indicate full length S and cleaved spike (S2 subunit), respectively. (D) SARS‐CoV-2 pseudovirus infection in Calu‐3 cells with or without treatment of coronavirus fusion peptide EK1. Luciferase activity was measured to quantify the infection. (E) SARS‐CoV-2 pseudovirus infection in Calu‐3 cells transduced with non‐targeting control or ACE2‐specific sgRNA. Luciferase activity was measured to quantify the infection. Mean ± SD of *n* = 3. ***P < 0.001, ****P < 0.0001, by Student's *t*‐test.F, GOverexpression of *CH25H* restricts SARS‐CoV-2 entry. Calu‐3 cells transduced with lentivirus overexpressing *CH25H* or empty vector were infected with SARS‐CoV-2 pseudovirus encoding Fluc or EGFP and pseudovirus infection was quantified by luciferase assay (F) or visualized by fluorescence microscopy (G). Scale bar, 100 μm. Mean ± SD of *n* = 3. ****P < 0.0001, by Student's *t*‐test. Source data are available online for this figure.

**Figure EV1:**
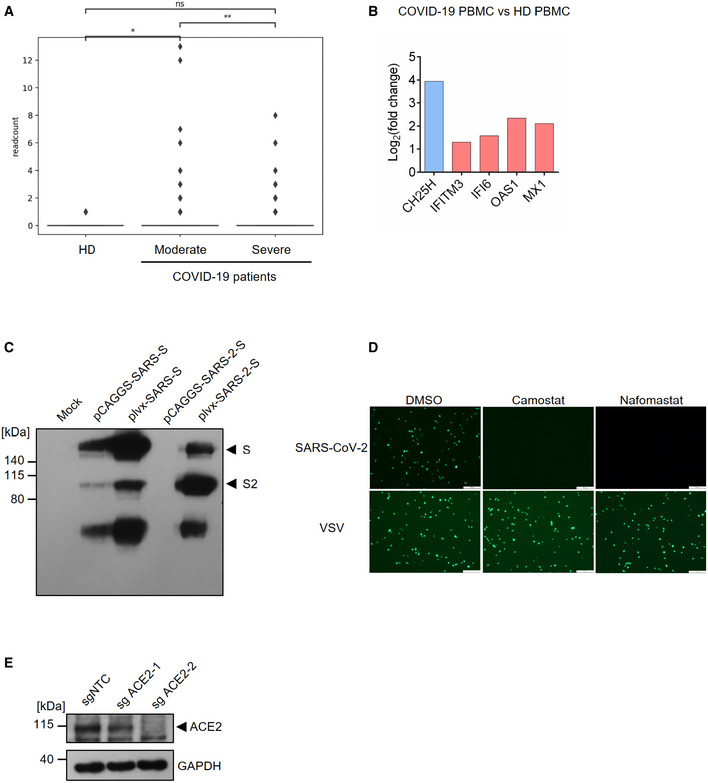
(Related to Fig 1). *CH25H* induction in COVID‐19‐infected patient and characterization of SARS‐CoV‐2 pseudovirus The box plot shows the expression of *CH25H* in epithelia of bronchoalveolar lavage fluids from four healthy donors, three moderate COVID‐19-infected patients and six severe COVID‐19-infected patients by scRNA‐seq analysis (Liao *et al*, 2020). *P < 0.05, **P < 0.01, by Student's *t*‐testRNA‐seq analysis showed robust induction of *CH25H* in PBMCs from COVID‐19-infected patients relative to healthy donors (Blanco‐Melo *et al*, 2020).Overexpression of SARS‐CoV-2 and SARS‐CoV spike protein in 293FT cells. The plasmids encoding SARS‐CoV-2 or SARS‐CoV spike protein was transfected in 293FT cells and expression of spike protein was analyzed by Western blotting using an antibody recognizing S2 subunit of SARS‐CoV and SARS‐CoV-2. Black arrows indicate full length spike protein and cleaved spike (S2 subunit), respectively.TMPRSS2 inhibitors, camostat and nafamostat, blocked entry of SARS‐CoV-2 but not VSV. Calu‐3 cells were treated with 10 μM camostat, 10 μM nafamostat or DMSO for 1 h. Then, the cells were infected with SARS‐CoV-2 or VSV pseudovirus with EGFP for 1 h. EGFP signals were captured by fluorescence microscope 24 h post‐infection. Scale bar, 100 μM.Knockout efficiency of ACE2 in Calu‐3 cells. Calu‐3 cells were transduced with non‐targeting control or ACE2‐specific sgRNA. ACE2 expression was analyzed by Western blotting. Black arrow indicates the band for ACE2. Source data are available online for this figure.

Since *CH25H* is an ISG and broadly inhibits viruses (Liu *et al*, 2013), we sought to investigate the role of CH25H during SARS‐CoV‐2 infection. We analyzed RNA‐seq data of SARS‐CoV‐2 infection in two lung epithelial cell lines, Calu‐3 and A549‐ACE2 (Blanco‐Melo *et al*, 2020), for up‐regulated ISGs. SARS‐CoV‐2 infection dramatically induced type I and III interferons, including IFNB1 and IFNL1, but not type II interferon (Fig 1A). Multiple well‐known ISGs were simultaneously elevated, such as *ISG15*, *OASL*, *IFITMs,* and the *IFITs* (Fig 1A). Importantly, *CH25H* expression was significantly up‐regulated in both cell lines (Fig 1A). Similar results were obtained from infections by human parainfluenza virus type 3 (HPIV3) and respiratory syncytial virus (RSV) but not influenza A virus, whose NS1 protein could completely block interferon pathways (Fig 1A). In corroboration with these cell line‐based data, scRNA‐seq analysis of bronchoalveolar lavage fluids from healthy donors and COVID‐19‐infected patients revealed an up‐regulation of *CH25H* in macrophages and epithelial cells in COVID‐19‐infected patients compared to healthy donors (Figs 1B and EV1A; Liao *et al*, 2020). Another RNA‐seq analysis also showed robust induction of *CH25H* in PBMCs from COVID‐19‐infected patients relative to healthy donors (Fig EV1B; preprint: Daamen *et al*, 2020).

Considering that CH25H has been demonstrated to restrict entry of several viruses including HIV, HSV, and a positive strand virus ZIKV (Liu *et al*, 2013; Li *et al*, 2017), we constructed SARS‐CoV‐2 pseudovirus based on VSV to investigate the viral entry process. We first checked the expression SARS‐CoV‐2 spike during packaging of VSV pseudovirus bearing spike (Fig EV1C). Immunoblotting revealed two forms of the spike protein: full length S and the S2 subunit (Fig 1C). Consistent with previous findings, S2 is highly abundant in SARS‐CoV‐2 (Fig 1C), because of a furin cleavage motif near the boundary of S1/S2 that promotes proteolytic processing (Hoffmann *et al*, 2020a). Similar bands were observed for VSV particles bearing the SARS‐CoV spike protein (Fig 1C), though the S2 band was less prominent due to the lack of any furin cleavage site (Hoffmann *et al*, 2020a). In addition, utilizing a fusion inhibition peptide EK1 targeting the HR1 domain of the spike protein, we confirmed that pseudovirus entry is mediated by the spike protein because EK1 treatment potently inhibited viral infection in Calu‐3 cells (Fig 1D). To further authenticate our pseudovirus, we tested infection of the human lung epithelial cell line Calu‐3 treated with TMPRSS2 inhibitors camostat and nafamostat (Fig EV1D). SARS‐CoV‐2 pseudovirus infection was abolished in cells treated with inhibitors as compared with vehicle control (Fig EV1D). Furthermore, infection was significantly repressed in ACE2 knockout Calu‐3 cells (Figs 1E and EV1E). Altogether, our results demonstrate that the entry of SARS‐CoV‐2 pseudovirus is mediated by spike and primed by cellular ACE2 and TMPRSS2, providing the validation of our pseudovirus system to authentically mimic SARS‐CoV‐2 entry.

Next, using the SARS‐CoV‐2 pseudovirus, we aimed to functionally validate the correlation between the up‐regulation of *CH25H* and SARS‐CoV‐2 infection. We overexpressed *CH25H* in Calu‐3 cells prior to SARS‐CoV‐2 pseudovirus challenge (Fig 1F and G). Our results showed that overexpression of *CH25H* significantly suppressed SARS‐CoV‐2 pseudovirus infection (Fig 1F and G). Taken together, these data suggest that the up‐regulation of *CH25H* upon SARS‐CoV‐2 infection *in vitro* and *in vivo* restricts SARS‐CoV‐2 infection.

### 25‐Hydroxycholesterol (25HC) broadly inhibits viral entry of human coronaviruses by blocking membrane fusion

**Figure 2:**
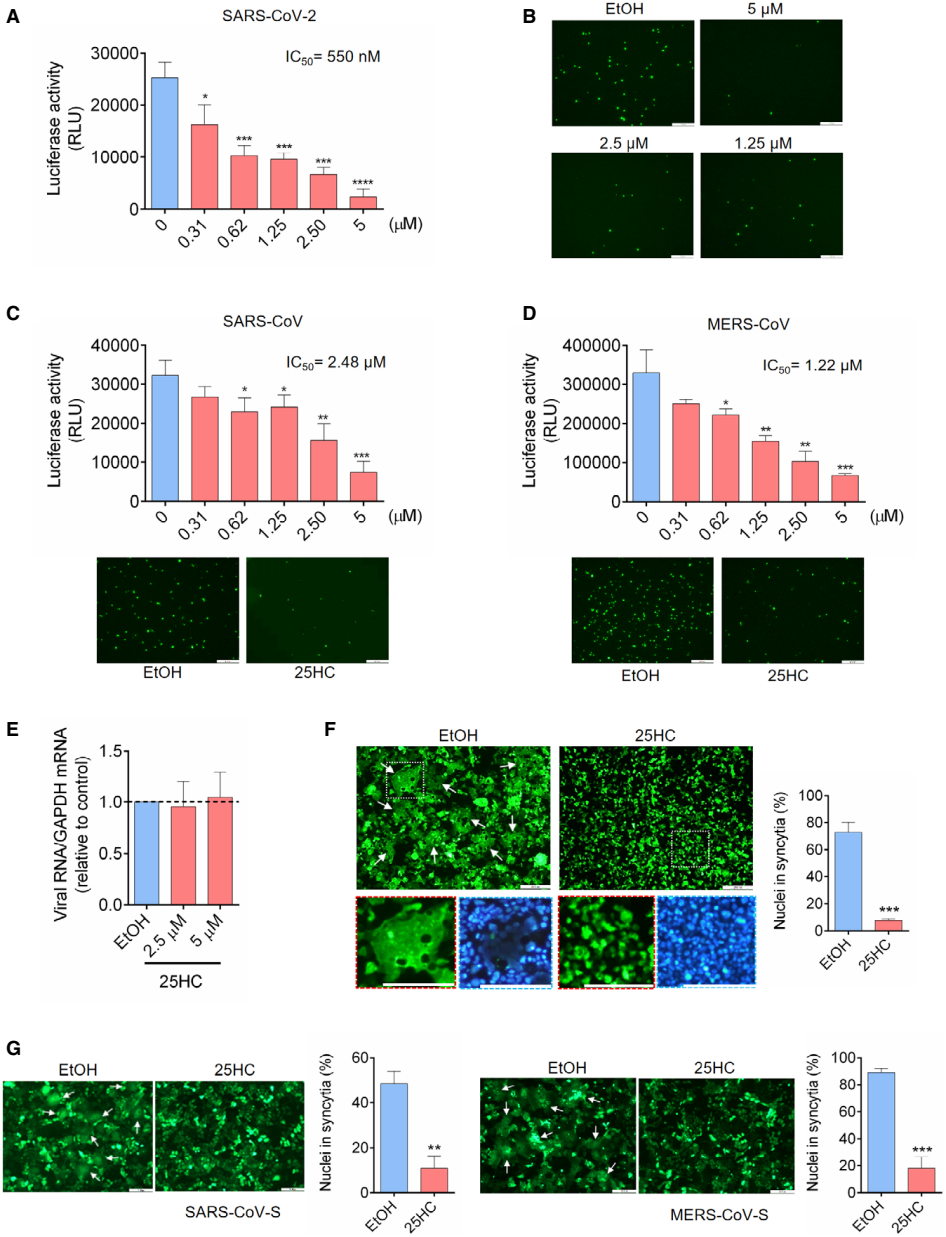
25‐Hydrocholesterol (25HC) blocks entry of human coronaviruses and spike‐mediated membrane fusion A–D25HC inhibits entry of SARS‐CoV-2 pseudovirus (A and B), SARS‐CoV pseudovirus (C), and MERS‐CoV pseudovirus (D). Calu‐3 cells were treated with EtOH or indicated concentrations of 25HC for 16 h. Cells were then challenged with FLuc‐expressing pseudovirus (A, C and D) and EGFP‐expressing pseudovirus (B, C and D) at MOI = 0.2, with EtOH or 25HC. After 2 h of infection, virus was removed and pseudovirus infection was quantified by luciferase assay at 24 h post‐infection (A, C and D) or visualized by fluorescence microscopy (B, C and D). IC_50_ was calculated by GraphPad using non‐linear regression analysis. Statistical analyses were conducted by Student's *t*‐test. Scale bar, 100 μm. Bar represents mean ± SD of *n* = 4. *P < 0.05, **P < 0.01, ***P < 0.001, ****P < 0.0001.E25HC does not inhibit SARS‐CoV-2 pseudovirus receptor binding. Calu‐3 cells were treated with EtOH or 25HC (2.5 or 5 μM) for 24 h prior to challenge by SARS‐CoV-2 pseudovirus at 4°C for 1 h. Total cellular RNA was extracted and quantified for VSV‐L by qPCR. Bar represents mean ± SD of *n* = 4.F, G25HC inhibits membrane fusion mediated by SARS‐CoV-2 S (F), SARS‐CoV S (G, left), and MERS‐CoV S (G, right). 293FT or Vero cells were treated with EtOH or 25HC (5 μM) for 16 h prior to transfection with pLVX plasmids encoding SARS‐CoV-2 S (F), SARS‐CoV S (G, left), and MERS‐CoV S (G, right) in the absence of EtOH or 25HC. At 24 h post‐transfection, syncytium formation was visualized by fluorescence microscopy. Scale bar, 50 μm. White arrows indicate syncytia. Quantification of membrane fusion was performed by calculating the percentages of nuclei involved in syncytia formation from all nuclei in GFP‐positive cells. Bar represents mean ± SD of *n* = 3. **P < 0.01, ***P < 0.001, by Student's *t*‐test.

**Figure EV2:**
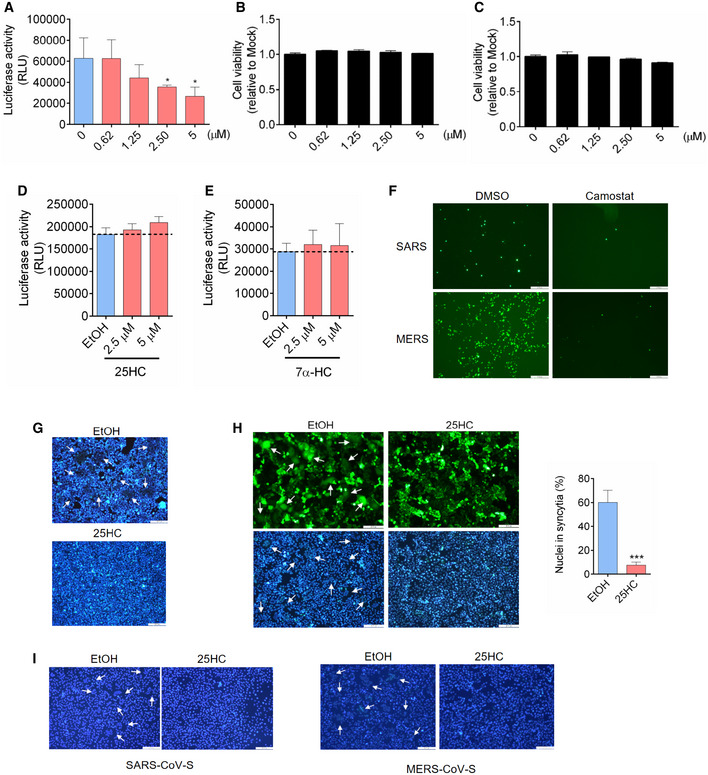
(Related to Fig 2). 25HC is not cytotoxic to Caco‐2 and Calu‐3 cells and inhibits coronavirus S‐induced membrane fusion A25HC inhibits SARS‐CoV-2 pseudovirus entry into Caco‐2 cells. Following a 16‐h incubation with EtOH or indicated concentrations of 25HC, cells were challenged with SARS‐CoV-2 pseudovirus encoding FLuc. After virus removal at 2 h, cells were lysed and luciferase activity was measured after 24 h. Statistical analyses were conducted by Student's *t*‐test. Bar represents mean ± SD of *n* = 3. *P < 0.05.B, C25HC is not cytotoxic to Caco‐2 (B) or Calu‐3 cells (C). Cell were treated with EtOH or indicated concentrations of 25HC for 24 h, and cell viability was measured by CellTiter‐Glo (Promega). Bar represents mean ± SD of *n* = 3.D25HC does not affect VSV genome replication. Cell were infected with VSV pseudovirus encoding FLuc for 2 h. Then, medium with EtOH or 25HC was added. At 24 h post‐infection, cells were lysed, and luciferase activity was measured. VSV replication was quantified by luciferase assays. Bar represents mean ± SD of *n* = 3.E7α‐HC does not inhibit SARS‐CoV-2 entry. Following a 16‐h incubation with EtOH or indicated concentrations of 7α‐HC, cells were challenged with SARS‐CoV-2 pseudovirus encoding FLuc for 2 h. At 24 h post‐infection, cells were lysed, and luciferase activity was measured. Statistical analyses were conducted by Student's *t*‐test. Bar represents mean ± SD of *n* = 3. *P < 0.05.FTMPRSS2 inhibitors camostat blocked entry of SARS‐CoV and MERS‐CoV pseudoviruses. Calu‐3 cells were treated with 10 μM camostat or DMSO for 1 h. Then the cells were infected with SARS‐CoV or MERS‐CoV pseudovirus with EGFP for 1 h. EGFP signals were captured by fluorescence microscope 24 h post‐infection. Scale bar, 100 μmGHoechst stain of nuclei in Fig 2F. White arrows indicate syncytia. Scale bar, 50 μm.H25HC inhibits cell–cell fusion on Vero cells. Vero cells were treated with EtOH or 25HC (5 μM) for 16 h prior to transfection with pLVX plasmids encoding SARS‐CoV-2 S. After 4 h, medium was changed and re‐supplemented with EtOH or 25HC. At 48 h post‐transfection, syncytium formation was visualized after trypsin treatment by fluorescence microscopy. White arrows indicate syncytia. Scale bar, 50 μm. Bar represents mean ± SD of *n* = 3. ***P < 0.001, by Student's *t*‐test.IHoechst stain of nuclei in Fig 2G. White arrows indicate syncytia. Scale bar, 50 μm.

To determine whether *CH25H* inhibits SARS‐CoV‐2 infection by 25HC production, Calu‐3 cells were treated with a concentration gradient of 25HC, followed by infection with SARS‐CoV‐2 pseudovirus encoding either Firefly luciferase or EGFP (Fig 2A and B). Pseudovirus entry was potently inhibited by 25HC in a dose‐dependent manner, with a half‐maximal inhibitory concentration (IC_50_) of 550 nM (Fig 2A). This inhibitory effect was confirmed by diminished numbers of EGFP‐positive cells, pretreated with 25HC as compared with ethanol (EtOH) vehicle, and challenged with EGFP‐expressing pseudovirus (Fig 2B). In light of the findings of SARS‐CoV‐2 infection in the gastrointestinal tract (Zang *et al*, 2020), we asked whether the viral inhibition by 25HC was specific to lung epithelial cells or it was a broad cell type mechanism for viral entry. To address this question, we analyzed pseudovirus entry into Caco‐2 cells, a colorectal epithelial cell line, which also expresses ACE2 and TMPRSS2 as found in Calu‐3 cells. Using FLuc‐expressing pseudovirus, we observed a dose‐dependent restriction of entry by 25HC (Fig EV2A). All concentrations of 25HC used in these experiments are non‐cytotoxic to both Caco‐2 (Fig EV2B) and Calu‐3 cells (Fig EV2C), and further 25HC does not exert significant background change in viral gene expression (Fig EV2D). In contrast, 7α‐HC, which is structurally similar to 25HC, did not show any antiviral activity (Fig EV2E). Thus, our results show that 25HC limits SARS‐CoV‐2 infection in both lung and intestinal epithelial cells.

We further tested restrictive potential of 25HC against two other human coronaviruses, SARS‐CoV and MERS‐CoV using VSV harboring SARS‐CoV S (Figs 2C and EV2F) and MERS‐CoV S (Figs 2D and EV2F). Entry of SARS‐CoV and MERS‐CoV pseudoviruses into Calu‐3 cells was suppressed by 25HC in a dose‐dependent manner as shown by luciferase assays, with IC_50_'s of 2.48 μM and 1.22 μM, respectively (Fig 2C and D, upper panels). This inhibition of infection was also confirmed by EGFP‐expressing pseudoviruses (Fig 2C and D, lower panels). These data, altogether, suggest that 25HC is an effective inhibitor of human coronaviruses entry.

To determine which step of SARS‐CoV‐2 entry is restricted by 25HC, we examined the effect of 25HC on SARS‐CoV‐2 pseudovirus receptor binding and fusion with the plasma membrane. Binding assays were performed by treating Calu‐3 cells with 25HC prior to SARS‐CoV‐2 pseudovirus challenge at 4°C to prevent internalization, and the amount of bound virions after three washes was quantified by RT–qPCR (Fig 2E). Our data indicate that 25HC does not significantly affect virus binding to the host cells (Fig 2E).

Next, we investigated the effect of 25HC on virus fusion with target cells. To simulate viral envelope fusion with plasma membrane, we performed cell–cell fusion studies by ectopically expressing SARS‐CoV‐2 S in EtOH‐ or 25HC‐treated 293FT cells (Figs 2F and EV2G) and Vero E6 cells (Fig EV2H) with EGFP to visualize spike‐induced syncytia (Belouzard *et al*, 2009). EtOH‐treated cells, but not 25HC‐treated cells, formed large multinucleate syncytia upon SARS‐CoV‐2 S expression (Figs 2F and EV2H). Since 25HC demonstrates broad anti‐human coronavirus activity, we asked whether 25HC could inhibit SARS‐CoV‐S‐mediated and MERS‐CoV‐S‐mediated cell–cell fusion. Similar to SARS‐CoV‐2 S, ectopic expression of SARS‐CoV‐S and MERS‐CoV‐S in EtOH‐treated, but not 25HC‐treated cells, induced the formation of large syncytia (Figs 2G and EV2I). These results strongly support a function for 25HC in blocking human coronavirus spike‐mediated fusion with the plasma membrane, thereby suppressing virus entry.

### 25HC blocks coronavirus entry and spike‐mediated membrane fusion through mobilizing accessible cholesterol from the plasma membrane

**Figure 3:**
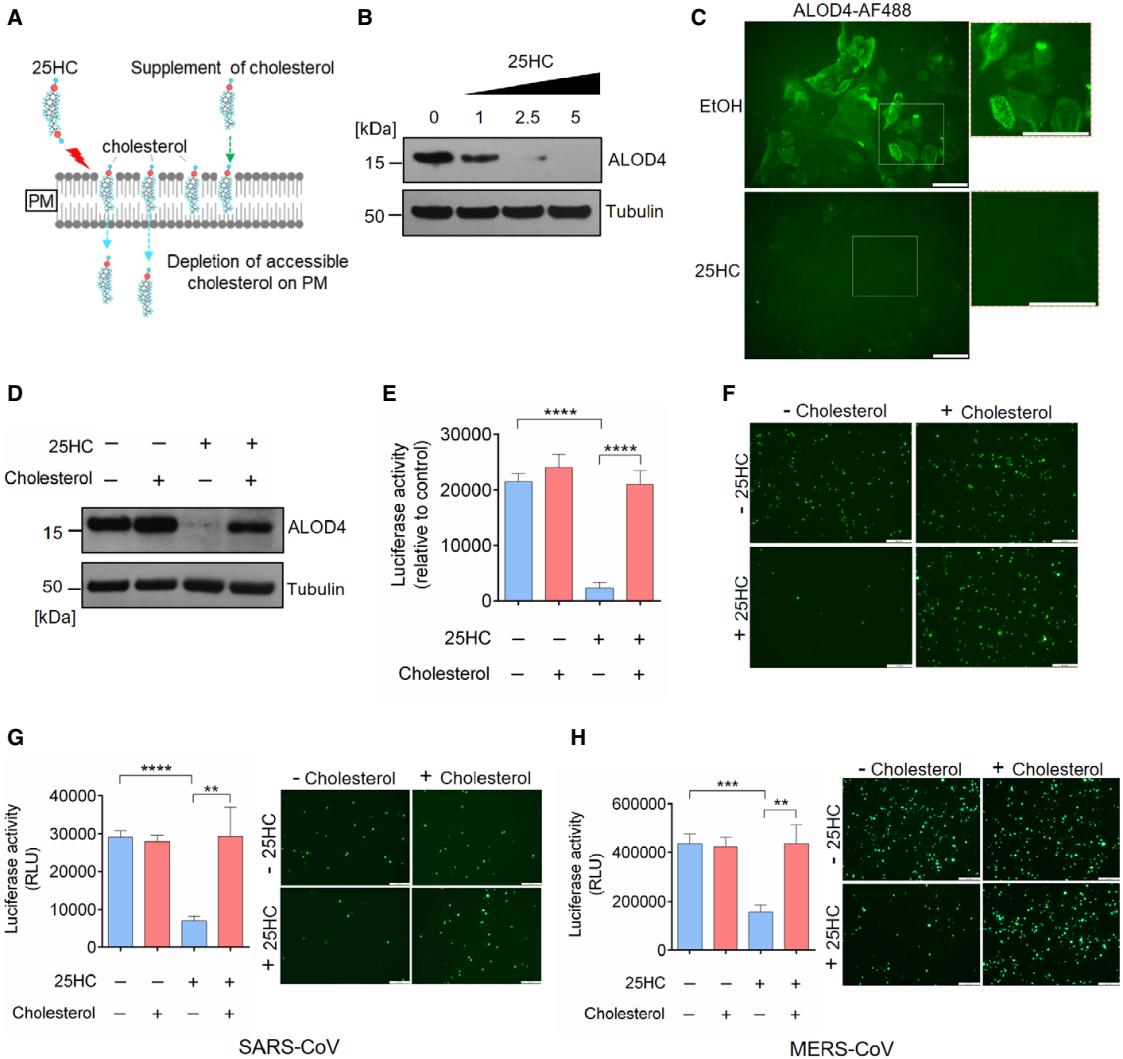
25‐Hydrocholesterol (25HC) inhibits viral entry through depleting accessible cholesterol on plasma membrane ARescue of cholesterol depletion on the plasma membrane (PM) reverses SARS‐CoV-2 entry block in Calu‐3 cells. 25HC induces the depletion of accessible cholesterol on the plasma membrane and supplement of additional cholesterol can reverse the depletion and viral entry.B, C25HC depletes accessible cholesterol on the plasma membrane. (B) Calu‐3 cells were treated with indicated concentrations of 25HC for 1 h and subsequently incubated with ALOD4 for 30 min. Cell were washed twice with PBS, lysed, and the amounts of bound ALOD4 were examined by Western blotting. (C) Calu‐3 cells were treated with 5 μM 25HC for 1 h, and subsequently incubated with AF488‐labeled ALOD4 for 30 min. Cells were washed twice and examined by fluorescence microscopy. Scale bar, 50 μm.DCholesterol rescues the level of accessible cholesterol on the plasma membrane of 25HC‐treated cells. Calu‐3 cells were pretreated with ethanol or 5 μM 25HC for 1 h. Cells were washed and incubated with PBS or 20 μM cholesterol for 1 h. After further incubation with ALOD4, cells were washed with PBS twice and lysed. The amount of bound ALOD4 was examined by Western blotting.E–HAddition of cholesterol rescues entry of SARS‐CoV-2 pseudovirus, SARS‐CoV pseudovirus, and MERS‐CoV pseudovirus into 25HC‐treated Calu‐3 cells. Cells were pretreated with ethanol or 5 μM 25HC for 16 h, washed, and incubated with PBS or 80 μM cholesterol for 1 h prior to infection with SARS‐CoV-2 pseudovirus. Entry of SARS‐CoV-2 pseudovirus (E and F), SARS‐CoV pseudovirus (G), and MERS‐CoV pseudovirus (H) was assessed by luciferase assay or by fluorescence microscopy at 24 h post‐infection. Scale bar, 100 μm. Bar represents mean ± SD of *n* = 3. **P < 0.01, ***P < 0.001, ****P < 0.0001, by Student's *t*‐test. Source data are available online for this figure.

**Figure EV3:**
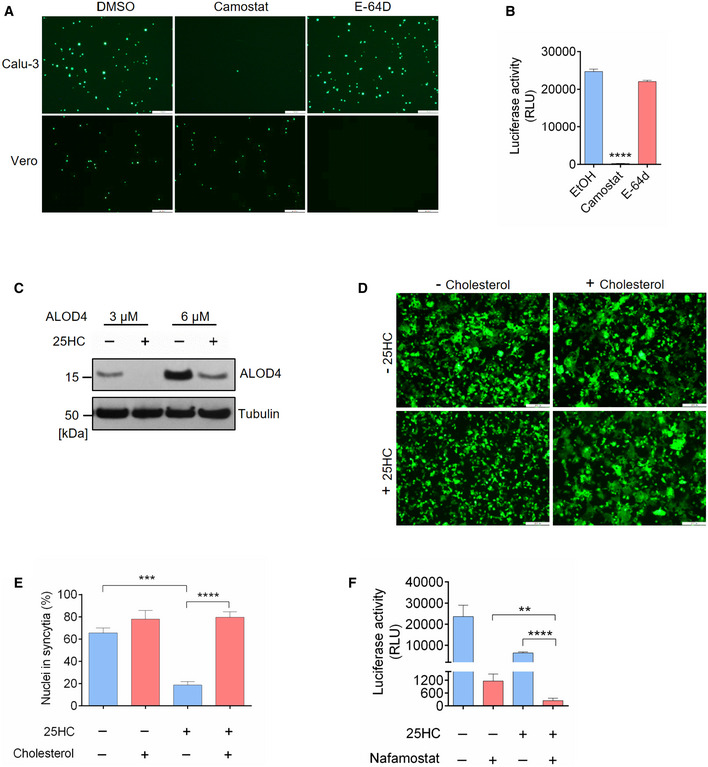
(related to Fig 3). Rescue of cholesterol depletion on the plasma membrane reverses SARS‐CoV‐2 spike‐mediated cell–cell fusion A, BSARS-CoV‐2 entry is dependent on TMPRSS2 but not endosomal cathepsin in lung epithelial cells. Calu‐3 cells were treated with 10 μM camostat or 25 μM E‐64D for 1 h. Then, the cells were infected with SARS‐CoV-2 pseudovirus with EGFP (A) or Fluc (B) for 1 h. EGFP signals were captured by fluorescence microscope 24 h post‐infection. Scale bar, 100 μm. Luciferase activity was measured at 24 h post‐infection. Bar represents mean ± SD of *n* = 3. ****P < 0.0001, by Student's *t*‐test.C25HC depletes accessible cholesterol on the plasma membrane. Calu‐3 cells were treated with EtOH or 5 μM 25HC and subsequently incubated with 3 or 6 μM ALOD4 for 30 min. The amount of bound ALOD4 was examined by Western blotting.D, ESupplement of cholesterol can rescue cell–cell fusion induced by SARS‐CoV-2 S. (D) 293FT cells were treated with EtOH or 25HC (5 μM) for 16 h prior to transfection with pLVX plasmids encoding SARS‐CoV-2 S. 4 h post‐transfection, medium was changed and re‐supplemented with EtOH or 25HC in the presence or absence of 80 μM cholesterol. 24 h post‐transfection, syncytium formation was visualized by fluorescence microscopy. Scale bar, 50 μm. (E) Membrane fusion was quantified as the percentage of nuclei within syncytia out of all nuclei in GFP‐positive cells. Bar represents mean ± SD of *n* = 3, ***P < 0.001, ****P < 0.0001, by Student's *t*‐test.FSynergistic effects of 25HC and TMPRSS2 inhibitor to block SARS‐CoV-2 entry. Addition of Nafamostat in 25HC‐treated cells were infected with SARS‐CoV-2 pseudovirus and 24 h post‐infection, cells were lysed, and luciferase activity was measured. Statistical analyses were conducted by Student's *t*‐test. Bar represents mean ± SD of *n* = 3. **P < 0.01, ****P < 0.0001. Source data are available online for this figure.

25HC and other oxysterols are important modulators of cholesterol metabolism and homeostasis (Du *et al*, 2004). Previous studies have demonstrated that 25HC changes the orientation and solvent accessibility of cholesterol and trigger cholesterol trafficking from the plasma membrane to the endoplasmic reticulum (Du *et al*, 2004). Intriguingly, a recent study on *Listeria* infection of epithelial cells reveals that 25HC restricts *Listeria monocytogenes* cell‐to‐cell dissemination through mobilizing cholesterol molecules free of sequestration by proteins and lipids from the plasma membrane (Abrams *et al*, 2020). These cholesterol molecules constitute a distinct accessible cholesterol pool (Das *et al*, 2014). Considering that 25HC blocked human coronavirus fusion at the plasma membrane of lung epithelial cells, we hypothesized that 25HC could inhibit SARS‐CoV‐2 entry through depleting accessible cholesterol on the plasma membrane (Fig 3A). Since we postulate a viral entry mechanism at the plasma membrane and not via endosome, we first confirmed that SARS‐CoV‐2 enters lung epithelial cells primarily through fusion at the plasma membrane and not by endocytosis. We analyzed viral entry in Calu‐3 and Vero cells in the presence of inhibitors for TMPRSS2 and Cathepsin B/L and compared with DMSO control. We found that camostat mesylate, a TMPRSS2 inhibitor, dramatically reduced viral entry in Calu‐3 cells and not in Vero cells (Fig EV3A and B). And cathepsin B/L inhibitor E‐64d efficiently blocked viral entry in Vero cells without effecting Calu‐3 infection (Fig EV3A and B). Therefore, the priming of spike triggering viral fusion is mediated by TMPRSS2 on the plasma membrane but not endosomal Cathepsin B/L. These results are consistent with previous findings that SARS‐2 S‐driven entry requires TMPRSS2 in Calu‐3 cells, which likely directs the virus to an early route of entry through fusion at the plasma membrane, and endosomal cathepsins in Vero cells, which are associated with a late route of entry through fusion in the endosome (Hoffmann *et al*, 2020b).

Next, we determined if 25HC could deplete accessible cholesterol on the plasma membrane of Calu‐3 cells by incubating cells with domain 4 of anthrolysin O (ALOD4), a bacterial toxin that specifically traps accessible cholesterol at the plasma membrane (Das *et al*, 2014; Abrams *et al*, 2020). 25HC depletes accessible cholesterol at the plasma membrane in a dose‐dependent manner (Figs 3B and EV3C), which corroborated with the loss of fluorescence signal from AF488‐labeled ALOD4 due to 25HC treatment (Fig 3C). To determine whether accessible cholesterol is essential for SARS‐CoV‐2 entry, we performed a functional cholesterol rescue experiment. We supplemented 25HC‐treated cells with exogenous cholesterol in cyclodextrin complex. We found that the depletion of accessible cholesterol could be rescued by supplementing soluble cholesterol to the cell culture (Fig 3D). If accessible cholesterol on the plasma membrane regulated by 25HC is essential for viral fusion, the replenishment of cholesterol should rescue viral fusion and lead to virus entry. As expected, supplementing cholesterol in 25HC‐treated cells rescued SARS‐CoV‐2 fusion and entry, but had no effect on vehicle‐treated cells (Figs 3E and F, and EV3D and E). These results provide evidence that 25HC mobilizes plasma membrane cholesterol to inhibit SARS‐CoV‐2 virus–cell fusion.

Given that 25HC inhibits viral entry of SARS‐CoV and MERS‐CoV (Fig 2C and D), we asked whether such inhibition was achieved through the same mechanism as described above for SARS‐CoV‐2. To address this question, Calu‐3 cells were pretreated with ethanol or 25HC and challenged with SARS‐CoV or MERS‐CoV pseudoviruses with or without a prior supplement of cholesterol. Addition of cholesterol reversed the inhibitory function of 25HC on viral entry for both SARS‐CoV and MERS‐CoV pseudoviruses (Fig 3G and H). Altogether, our results show that 25HC restricts coronavirus entry by depleting accessible cholesterol on the plasma membrane.

### 25HC depletes accessible cholesterol on the plasma membrane through activating acyl‐CoA:cholesterol acyltransferase (ACAT)

**Figure 4:**
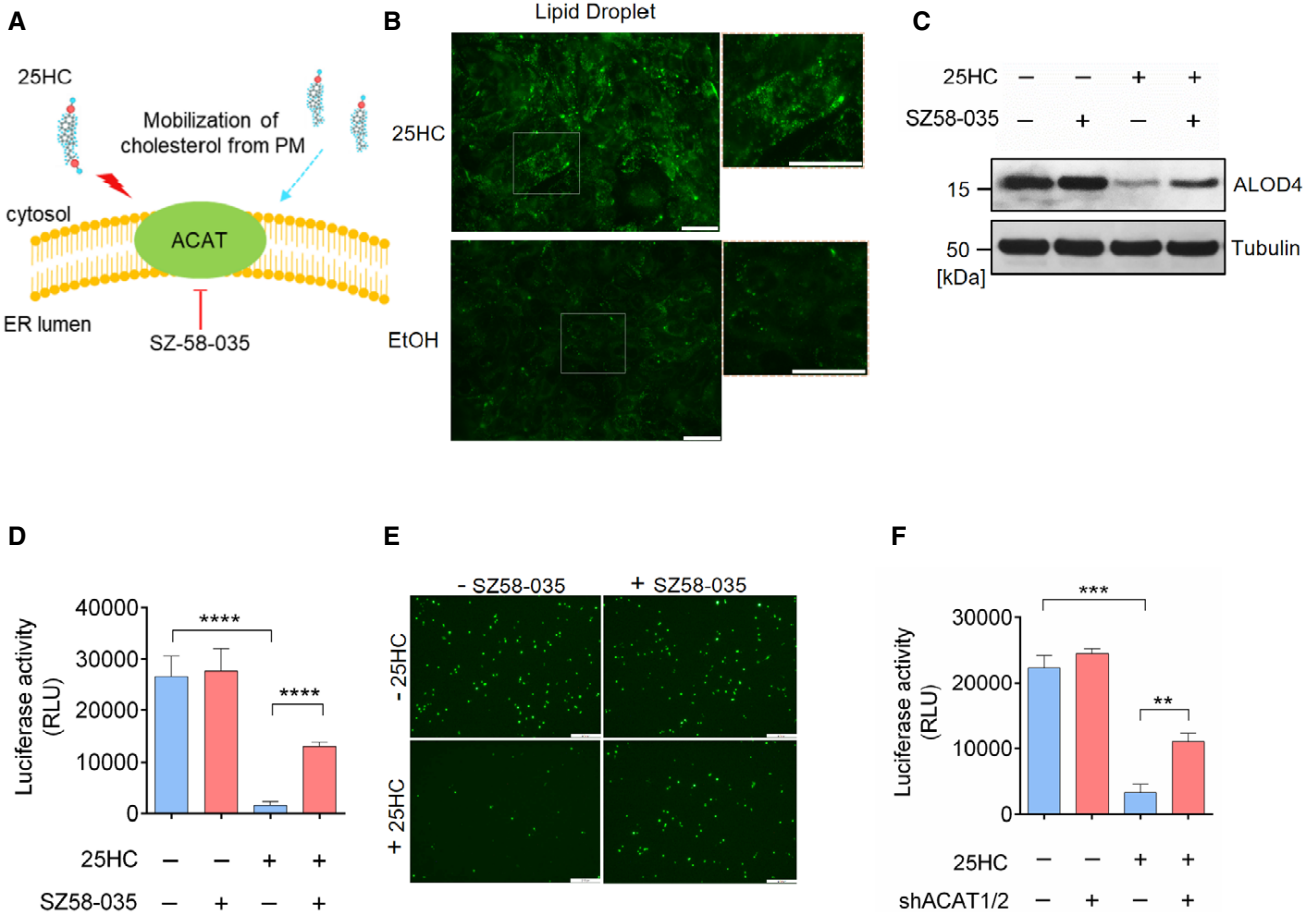
25‐Hydrocholesterol (25HC) activates acyl‐CoA:cholesterol acyltransferase (ACAT) and induces internalization of accessible cholesterol from the plasma membrane AInhibition of cholesterol mobilization from the plasma membrane by the ACAT inhibitor SZ58‐035.B25HC activates ACAT and stimulates the formation of lipid droplets. Calu‐3 cells were cultured in medium containing 2% LPDS prior to the treatment of ethanol or 5 μM 25HC for 16 h. Subsequently, cells were stained by LipidSpot to visualize intracellular lipid droplets. Scale bar, 50 μm.CSZ58‐035 rescues surface accessible cholesterol in 25HC‐treated cells. Calu‐3 cells were treated with vehicle or 40 μM SZ58‐035 for 16 h, prior to treatment with ethanol or 2.5 μM 25HC for 1 h. Cells were then incubated with ALOD4 for 30 min. The amount of bound ALOD4 was examined by Western blotting.D, EInhibition of ACAT activity by SZ58‐035 rescues viral entry viral entry. Calu‐3 cells were pretreated with ethanol or 5 μM 25HC for 16 h in the presence of DMSO or 40 μM SZ58‐035. Then, cells were washed and incubated with DMSO or 40 μM SZ58‐305 for 1 h prior to infection with SARS‐CoV-2 pseudovirus. Luciferase activity was measured at 24‐hour post‐infection (D). EGFP signal was captured by fluorescence microscope 24 h post‐infection (E). Scale bar, 100 μm. Bar represents mean ± SD of *n* = 3. ****P < 0.0001, by Student's *t*‐test.FKnockdown of ACAT1/2 rescues SARS‐CoV-2 pseudovirus entry in 25HC‐treated cells. Calu‐3 cells were transduced with lentiviral vectors carrying non‐targeting shRNA or shRNAs targeting ACAT1 and ACAT2. 48 h post‐transduction, cells were treated with ethanol or 5 μM 25HC for 16 h. Then, the cells were challenged with SARS‐CoV-2 pseudovirus. 24 h post‐pseudovirus challenge, viral entry was quantified by luciferase assays. Bar represents mean ± SD of *n* = 3. **P < 0.01, ***P < 0.001, by Student's *t*‐test. Source data are available online for this figure.

**Figure EV4:**
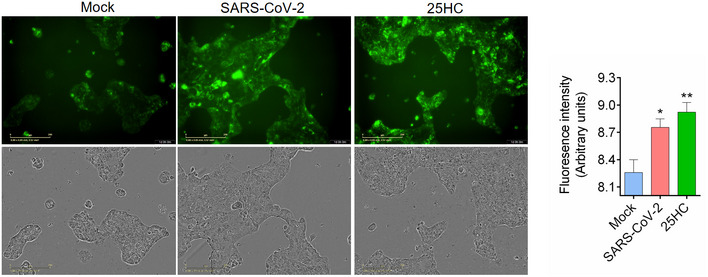
(related to Fig 4). SARS‐CoV‐2 isolate USA‐WA1/2020 activates ACAT Calu‐3 cells were infected with SARS‐CoV‐2 USA‐WA1/2020 at MOI = 2 for 24 h at 37°C or treated with 5 μM 25HC for 24 h. Cells were then stained with 1X LipidSpot 488 for 30 min. Fluorescence intensity was quantified by Incucyte S3 analysis software. Scale bar, 200 μm.

Next, we sought to elucidate the mechanism of cholesterol mobilization induced by 25HC. 25HC enhances cholesterol esterification and in turns trigger cholesterol transport from the plasma membrane to the ER (Du *et al*, 2004; Das *et al*, 2014). We hypothesized that the enzyme catalyzing the esterification of cholesterol, ACAT, can be activated by 25HC and SARS‐CoV‐2 to direct the trafficking of cholesterol from the plasma membrane. To test this hypothesis, we employed the selective ACAT inhibitor, Sandoz 58‐035 (SZ58‐035), to determine the role of ACAT in modulating accessible cholesterol and suppressing SARS‐CoV‐2 pseudovirus entry (Fig 4A). Since upon esterification by ACAT, cholesteryl esters are stored in cytosolic lipid droplets, we used a lipid droplet stain to monitor ACAT activity (Dong *et al*, 2020). We first confirmed that 25HC could activate ACAT by staining intracellular lipid droplets in vehicle‐ or 25HC‐treated Calu‐3 cells. We found a dramatic increase in lipid droplets in 25HC‐treated cells, indicating an increased ACAT‐mediated cholesterol esterification (Fig 4B). Next, we treated vehicle‐ or 25HC‐conditioned Calu‐3 cells with or without SZ58‐035 and analyzed accessible cholesterol at the plasma membrane by ALOD4. Our results showed that when ACAT was inhibited, accessible cholesterol was increased suggesting that SZ58‐35 stalls the depletion of accessible cholesterol by 25HC (Fig 4C). In accordance with these observations, SZ58‐035 treatment rescued SARS‐CoV‐2 pseudovirus entry (Fig 4D and E). Furthermore, we infected Calu‐3 cells with the SARS‐CoV‐2 isolate USA‐WA1/2020 strain and analyzed lipid droplet formation. Our results showed that authentic virus infection efficiently activated ACAT that was comparable to 25HC treatment (Fig EV4). To further define the specificity of ACAT inhibition and its effect on viral entry, we silenced ACAT by shRNAs and analyzed pseudovirus entry in the presence and absence of 25HC. We found that ACAT knockdown by shRNA enhanced pseudovirus entry into 25HC‐treated Calu‐3 cells compared to non‐targeting shRNA (Fig 4F), a finding similar to drug mediated inhibition of ACAT (Fig 4D).

Collectively, these results show that ACAT is activated in response to 25HC and triggers the mobilization of accessible cholesterol from the plasma membrane, thereby restricting SARS‐CoV‐2 entry.

### 25HC inhibits SARS‐CoV‐2 isolate USA‐WA1/2020 infection and viral entry in human lung organoids

**Figure 5:**
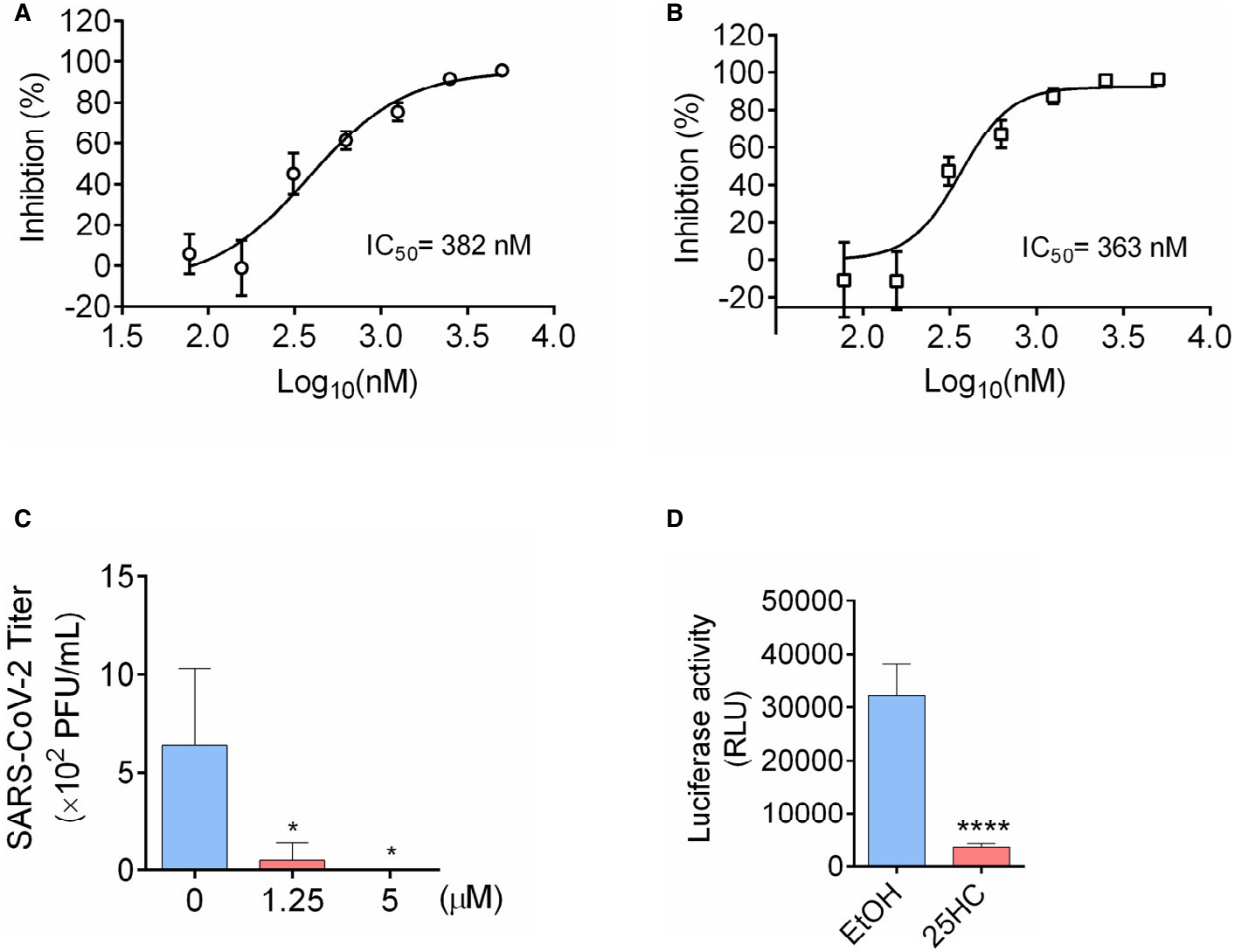
25‐Hydrocholesterol (25HC) inhibits SARS‐CoV‐2 isolate USA‐WA1/2020 infection and viral entry in human lung organoids A, B25HC inhibits SARS‐CoV-2 USA‐WA1/2020 strain infection in Calu‐3 cells. Cells were pretreated with ethanol or 25HC followed by infection. At 48 h post‐infection, RNA from supernatant (A) and infected cells (B) were extracted and quantified by qPCR. Data are mean ± SD of *n* = 3, representative of 3 repeats.CProgeny virus titer in supernatant was quantified by plaque assays. Data are mean ± SD of *n* = 3, representative of 3 repeats. *P < 0.05, by Student's *t*‐test.DTwo‐month‐old differentiated lung organoids were pretreated with 25HC (5 μM) for 16 h and infected with luciferase‐expressing SARS‐CoV-2 pseudovirus at MOI = 0.1 for 2 h and treated with fresh medium containing 5 μM 25HC. Luciferase activity was measured at 24 h post‐infection. Mean ± SD of *n* = 3. ****P < 0.0001, by Student's *t*‐test.

To further confirm the antiviral activity of 25HC during a patient isolated SARS‐CoV‐2 infection, Calu‐3 cells were treated with a concentration gradient of 25HC and SARS‐CoV‐2 USA‐WA1/2020 infection was quantified by evaluating viral RNA of the infected cells and their culture supernatants (Fig 5A and B). Viral infection was inhibited by 25HC in a dose‐dependent manner. The IC50 values, as determined by supernatant viral RNA and intracellular viral RNA, are 382 nM and 363 nM, respectively (Fig 4A and B), which are quite similar to the IC50 for the inhibition of SARS‐CoV‐2 pseudovirus infection in Calu‐3 cells (Fig 2A). Supernatants of infected 25HC‐treated cultures also contain significantly less virus progeny than vehicle control (Fig 5C). Because organoids are more physiologically relevant than cell lines as infection models, we next sought to confirm the function of 25HC using 3D lung organoids. Human iPSC derived lung organoids were generated using previously published methods (Leibel *et al*, 2019; Miller *et al*, 2019) and characterized by analyzing expression of ACE2, TMPRSS2, and alveolar cell epithelial markers (SFTPC, SFTPB, HOPX; Tiwari et al. Manuscript under preparation). These 60D differentiated lung organoids were pretreated overnight with 25HC (5 μM) and were challenged with luciferase‐expressing SARS‐CoV‐2 pseudovirus. After 24 h, luciferase activity was measured to quantify viral levels. 25HC treatment significantly blocked SARS‐CoV‐2 entry in lung organoids (Fig 5D). Altogether, results from lung epithelial cells and organoids further support that 25HC efficiently blocks SARS‐CoV‐2 entry in lung cells.

## Discussion

In this study, we characterized the anti‐SARS‐CoV‐2 function and mechanism of *CH25H*, an interferon‐inducible gene robustly up‐regulated in lung epithelial cell lines infected with SARS‐CoV‐2 and in COVID‐19‐infected patients. We further showed that 25HC, the product of the interferon‐inducible enzyme CH25H, possesses broad‐spectrum antiviral activity against human coronaviruses. Further mechanistic studies revealed viral membrane fusion was blocked by this oxysterol. Previous studies demonstrated that 25HC inhibited entry of various types of viruses including VSV, HIV, NiV, EBOV, and ZIKV (Liu *et al*, 2013; Li *et al*, 2017). However, the mechanism by which 25HC modulates viral entry is still unknown. In addressing the mechanism of 25HC function, we found that 25HC triggered the depletion of accessible cholesterol from the plasma membrane via activating ACAT, thereby leading to inhibition of virus entry. The ER‐localized enzyme ACAT uses fatty acyl‐CoA and cholesterol as substrates to produce cholesteryl esters for storage in cytoplasmic lipid droplets and induces the depletion of accessible cholesterol from the plasma membrane through unknown mechanisms (Abrams *et al*, 2020). Altogether, our results provide evidence for a functional link between SARS‐CoV‐2 induced up‐regulation of *CH25H* and the inhibition of SARS‐CoV‐2 entry by the CH25H product 25HC and reveal the broad‐spectrum antiviral mechanism of this oxysterol.

Sterols and oxysterols influence immune system and viral infections through both general and cell‐specific mechanisms (Spann & Glass, 2013). Cholesterol has multiple functions on lipid bilayers. An increase or decrease of cholesterol can be accompanied by changes in the fluidity, polarity, thickness, and intrinsic curvature of membrane. Changes in cholesterol could affect the function of integral membrane proteins, including viral receptor or co‐receptors, by altering their conformation or distribution on the plasma membrane (Yang *et al*, 2016). These alterations will directly or indirectly affect virus–cell membrane fusion, which is essential for viral genome release (Chazal & Gerlier, 2003). The depletion of cholesterol by 25HC did not affect viral binding and TMPRSS2 priming (Figs 2E and EV3F). It is possible the depletion of cholesterol directly affects virus–cell fusion. Given the importance of membrane cholesterol for virus–cell fusion, the mechanism of 25HC could possibly be extended to other viruses that fuse with a cellular membrane before entry. Indeed, cholesterol has been proved to be important for the fusion and post‐entry steps in the flavivirus life cycle (Osuna‐Ramos *et al*, 2018). Depletion of cholesterol blocked HIV fusion at the plasma membrane (Yang *et al*, 2015). Given that 25HC seems to inhibit the entry of multiple enveloped viruses, we speculate that enveloped viruses inhibited by 25HC at the fusion step utilize our proposed mechanism, suggesting a fundamental role for this small molecule in the innate immune system (Liu *et al*, 2013).

The COVID‐19 pandemic is spreading and causing great human loss and suffering, but no vaccine or effective therapeutic strategy is available. It is urgent to develop antiviral drugs against SARS‐CoV‐2. Statins are commonly used as lipid‐lowering drugs that also have anti‐inflammatory effects; therefore, use of statin has been suggested for COVID‐19 treatment (Castiglione *et al*, 2020; Dashti‐Khavidaki & Khalili, 2020). However, some studies suggest that the expression of ACE2 receptor, which is required for SARS‐CoV‐2 entry (Hoffmann *et al*, 2020b), is also up‐regulated by statins (Tikoo *et al*, 2015; Shin *et al*, 2017) raising concerns about the use of statin for COVID‐19 therapy. Intriguingly, a retrospective clinical study on 13,981 patients found that statin use was associated with reduced risk of mortality in COVID‐19‐infected patients (Zhang *et al*, 2020). Compared with statins, 25HC only decreases accessible cholesterol at the plasma membrane without altering the total cellular cholesterol; therefore, it may cause lower cytotoxicity or side effects than statin use. For example, we did not observe any significant increase in *ACE2* and *TMPRSS2* mRNA expression by 25HC treatment of Calu‐3 cells. In addition, 25HC is a natural product and has not shown toxicity in mice and non‐human primates at effective concentrations. Thus, 25HC has potential as an antiviral agent to combat with SARS‐CoV‐2 and other possible emerging coronaviruses in the future.

## Materials and Methods

### Cell culture and reagents

All studies were performed in accordance with approved IRB protocols by the University of California (UCSD), San Diego. Human lung epithelia cell line Calu‐3, human colon epithelia cell line Caco‐2 were obtained from ATCC. Calu‐3 was maintained in MEM medium supplemented with 1× NEAA, penicillin‐streptomycin (100 IU/ml), and 10% FBS. Caco‐2 was cultured in MEM medium with 1× NEAA, penicillin‐streptomycin (100 IU/ml) and 20% FBS. 293FT and Vero E6 cells were maintained in DMEM medium with 10% FBS. Spike antibody recognizing S2 subunit of SARS‐CoV and SARS‐CoV‐2 was purchased from Nouvs (NB100‐56578). Anti‐VSV M antibody was from EMD Millipore (MABF2347). EK1 peptides were obtained from Phoenix Pharma. Luciferase assay kit was purchased from Promega. 7α‐HC, 25HC, water‐soluble cholesterol, and Sandoz 58‐035 were obtained from Sigma. Camostat, nafamostat and E‐64d were purchased from Selleck Chemicals.

### VSV pseudovirus rescue, amplification and titration

Pseudo typed VSV was generated based on previously described work (Whitt, 2010). In brief, BHK‐21/WI‐2 cells were infected with vTF7‐3 expressing T7 polymerase for 45 min and then transfected with pVSV‐EGFP‐dG (addgene 31842) or pVSV‐FLuc‐dG, along with pBS vectors expressing VSV‐N, VSV‐P, VSV‐L, and VSV‐G (Kerafast) at ratio 3:3:5:1:8, using Opti‐MEM and Lipofectamine 3000 according to the manufacturers’ instructions. 48 h post‐transfection, culture supernatants were collected and filtered through 0.22 μm filters to remove residual vaccinia virus. 293FT cells transfected with pMD2.G (addgene 12259) encoding VSV‐G for 24 h were inoculated with the filtrate to amplify the VSV pseudovirus. 24 h post‐inoculation, supernatants were collected and either stored at −80°C. Titration of EGFP‐expressing pseudovirus was performed by inoculating Vero E6 cells seeded in 96‐well plates with 10‐fold serial dilutions of the virus stock. 24 h post‐inoculation, numbers of GFP‐positive cells were counted and used to calculate virus titer as infectious unit per milliliter (IU/ml). Titration of FLuc‐expressing pseudoviruses was performed by inoculating Vero E6 cells transfected with VSV‐G in 24‐well plates with 10‐fold serial dilutions of the virus stock. 1 h post‐inoculation, new medium containing 1.5% methylcellulose was used to replace the inoculum. 2 days post‐inoculation, cells were washed and stained with crystal violet. Plaque numbers were counted and used to calculate virus titer.

### SARS‐CoV‐2, SARS‐CoV, and MERS‐CoV pseudovirus production, titration, and characterization

293FT cells were transfected with pLVX expressing SARS‐CoV‐2‐S, SARS‐CoV‐S, or MERS‐CoV‐S. Nineteen amino acids at the C terminus of SARS‐CoV‐2‐S and SARS‐CoV‐S was deleted, given previous report that the shortened mutant has improved incorporation into the pseudovirus envelope (Fukushi *et al*, 2005; Hoffmann *et al*, 2020a). At 24 h post‐transfection, cells were infected with VSV pseudovirus containing Fluc or EGFP. Cells were washed four times with medium 1 h post‐inoculation and maintained in medium for 24 h. Then supernatant containing pseudovirus was collected, centrifuged, and used or stored at −80°C. The titer was measured using the same method for VSV pseudovirus mentioned above. To characterize the pseudovirus, 1 ml supernatant was concentrated by ultra‐centrifugation at 106,750 × *g* for 2 h at 4°C. Then, 40 μl lysate buffer with SDS was added and heated for 5 min at 95°C. The samples were subjected to SDS–PAGE and immunoblotting using spike antibody and VSV matrix antibody.

### SARS‐CoV‐2, SARS‐CoV, and MERS‐CoV pseudovirus infection and inhibitors treatment

Cells were pretreated with 25HC for 16 h and then infected with SARS‐CoV‐2, SARS‐CoV, and MERS‐CoV pseudovirus in fresh medium without 25HC. After 2‐h infection, virus was removed and fresh medium without 25HC was added. For pseudovirus with Fluc, cells were lysed at 24 h post‐infection and subjected for luciferase activity assay according to manufacturer's instruction. For pseudovirus with EGFP, infected was evaluated by taking picture for at least 5 random fields at 24 h post‐infection.

### Cell–cell fusion assay

293FT or Vero E6 cells were treated with EtOH or 25HC overnight, and then co‐transfected with pEGFP‐C1 and a pLVX vector expressing one of SARS‐CoV‐2‐S, SARS‐CoV‐S, and MERS‐CoV‐S using Lipofectamine 3000, in the absence of EtOH or 25HC. 4 h post‐transfection, fresh medium containing EtOH and 5 μM 25HC was supplemented. For 293FT cells, 24 h post‐transfection, cells were stained with Hoechst for 10 min at 37°C and examined under Leica fluorescence microscope (DMI 3000). For Vero E6 cell, 48 h post‐transfection, cells were washed twice with PBS, and incubated with 2 μg/ml Trypsin in PBS for 20 min. Trypsin‐containing PBS was subsequently removed, and cells were further incubated in medium for 40 min, prior to a 10‐min Hoechst staining at 37°C and examination under a fluorescence microscope. For quantification of membrane fusion, the percentage of nuclei involved in syncytia formation in each group was calculated by averaging three corresponding fields, and the extent of membrane fusion for each group was calculated by averaging 3 independent biological replicates for that group.

### Pseudovirus binding assay

Calu‐3 cells were treated with EtOH or indicated concentrations of 25HC for 16 h, prior to incubation with SARS‐CoV‐2 pseudovirus at MOI = 2 at 4°C. Cells were then washed with chilled PBS three times and lysed with TRIzol. The RNA was extracted using a Direct‐zol RNA Kit (Zymo). The amount of bound virions was measured by RT–qPCR using primers for VSV‐L. The primers are as follows: *GAPDH* (Forward: GGCCTCCAAGGAGTAAGACC; Reverse: AGGGGTCTACATGGCAACTG), *VSV*‐*L* (Forward: GACGGGCTCATCAGTCTATTT; Reverse: GGATACCTCACTCCTCACAATC).

### ALOD4 protein purification, labeling, and incubation

ALOD4 protein was expressed, purified, and labeled based on a previously described protocol (Endapally *et al*, 2019). Briefly, the plasmid pALOD4 (Addgene 111026) was transformed into BL21(DE3)pLysS competent cells (Invitrogen) (Gay *et al*, 2015). ALOD4‐His_6_ was purified using Capturem His‐tagged purification kit (Takara). The purified protein was further concentrated by 10 kD Amicon Ultra centrifugal filters. For ALOD4 labeling by Alexa Fluor 488 C5 (AF488) (Invitrogen), ALOD4 was mixed with AF488 at 4°C for 16 h. The reaction was quenched by 10 mM DTT. Excess dye was removed by desalting.

For ALOD4 binding assay, 3 μM purified or labeled ALOD4 was added to each well, and ethanol‐ or 25HC‐treated cells were incubated with ALOD4 for 30 min at 37°C. Cells were washed twice with PBS prior to WB sample preparation. For ALOD4 binding assay with cholesterol rescue, ethanol‐ and 25HC‐treated cells were treated with cholesterol for 1 h at 37°C prior to incubation with ALOD4. For ALOD4 binding assay with SZ58‐035, vehicle or SZ58‐035 was present during the treatment with 25HC and incubation with ALOD4.

### Cholesterol and Sandoz 58‐035 rescue assays

Calu‐3 cells were pretreated with Ethanol or 5 μM 25HC for 16 h. For cholesterol rescue experiment, the cells were washed and incubated with PBS or 80 μM water‐soluble cholesterol for 1 h. Fresh medium and SARS‐CoV‐2 pseudovirus without compounds were added to incubate with the cells for 2 h. Then, cells were washed and cultured in fresh medium. For Sandoz 58‐035 rescue experiment, after overnight treatment of 25HC in the presence of DMSO or 40 μM SZ58‐035, Calu‐3 cells were washed and incubated with DMSO or 40 μM SZ58‐035 for 1 h prior to infection with SARS‐CoV‐2 pseudovirus. For pseudovirus with Fluc, the cells were lysed and subjected to luciferase activity measurement at 24 h post‐infection. For EGFP pseudovirus, EGFP signals were captured by fluorescence microscope at 24 h post‐infection.

### ACAT knockdown rescue assays

Calu‐3 cells were transduced with lentivirus expressing shACAT1/2 for 48 h. Then, cells were treated with medium containing EtOH or 5 μM 25HC overnight. Calu‐3 cells were washed prior to infection with SARS‐CoV‐2 pseudovirus. The cells were lysed and subjected to luciferase activity measurement at 24 h post‐infection.

### SARS‐CoV‐2 isolate USA‐WA1/2020 infection

SARS‐CoV‐2 isolate USA‐WA1/2020 was obtained from BEI Resources. SARS‐CoV‐2 was propagated and infectious units quantified by plaque assay using Vero E6 cells. Calu‐3 cells were pretreated with 25HC overnight and then infected with SARS‐CoV‐2 at MOI = 0.1 for 1 h at 37°C. Then, cells were washed and fresh medium with 25HC was supplemented. 48 h post‐infection, supernatant and infected cells were lysed using TRIzol and RNA was extracted using a Direct‐zol RNA Kit (Zymo) and quantified by RT–qPCR using SARS‐CoV‐2 N primers. The primers are as follows: *SARS‐CoV-2‐N* (Forward: CACATTGGCACCCGCAATC; Reverse: GAGGAACGAGAAGAGGCTTG). Viral titer in supernatant was quantified by plaque assay in Vero cells.

### Lipid droplet staining

Calu‐3 cells were suspended in MEM medium with 2% lipid depleted serum and seeded in the wells of a Lab‐TEK II 4‐well chamber or a 96‐well plate. For the treatment with 25HC, cells were treated with 5 μM 25HC for 16 h. Then, the cells were washed and stained with 1× LipidSpot 488 (Biotium) for 30 min. The signals were captured by fluorescence microscope. For the infection with live SARS‐CoV‐2 virus, Calu‐3 cells were infected with SARS‐CoV‐2 USA‐WA1/2020 at MOI = 2 for 1 h at 37°C. Then, cells were washed, and fresh medium was supplemented. Cells were stained with 1× LipidSpot 488 for 30 min after 24 h post‐infection. The fluorescence signal was captured by Incucyte S3.

### Lung Organoid 25HC treatment and infection

Human iPSC derived lung organoids were generated using previously published methods (Leibel *et al*, 2019; Miller *et al*, 2019) and characterized by expression of ACE2, TMPRSS2 and alveolar cell epithelial markers (SFTPC, SFTPB, HOPX) (Tiwari *et al*, Manuscript under preparation). These 60D differentiated lung organoids were pretreated overnight with 25HC (5 μM) and were infected with luciferase‐expressing SARS‐CoV‐2 pseudovirus (100 μl). At 2 h post‐infection, organoids were incubated with fresh medium containing 25HC. After 24 h, luciferase activity was measured to quantify viral infection.

### Lentivirus production and transduction

Two sgRNA oligos targeting ACE2 were cloned into lentiCRISPR v2 (addgene 52961) and two shRNA oligos targeting ACAT1 and ACAT2 were cloned into pLKO.1 (addgene 8453). *CH25H* was cloned into pLVX vectors. The target sequences of sgRNA and shRNA are as follows: *ACE2*‐sgRNA1: CAGGATCCTTATGTGCACAA; *ACE2*‐sgRNA2: CCAAAGGCGAGAGATAGTTG; *ACAT1*‐shRNA: GCCACTAAGCTTGGTTCCATT; *ACAT2*‐shRNA: GCTCTTATGAAGAAGTCAGAA. The cloning primers for CH25H (Forward: GGATCTATTTCCGGTGAATTCATGAGCTGCCACAACTGCTCC; Reverse: GCGGTCATACGTAGGATCCTTACCGCGCTGGGACAGATG). 293FT cells seeded in 6‐well plates were co‐transfected with pMD2.G (0.6 μg), psPAX.2 (1.2 μg), and sgRNA, pLVX, or pLKO.1 vector (1.8 μg) using Opti‐MEM and Lipofectamine 3000 according to the manufacturers’ instruction. At 4 h post‐transfection, supernatant was replaced with fresh medium. At 48 h post‐transfection, supernatant was harvested and used to transduce Calu‐3 cells. After 12 h transduction, lentivirus was removed and replaced with fresh medium.

### Cell viability assay

Calu‐3 or Caco‐2 cells were treated with EtOH or twofold serial dilutions of 25HC for 48 h. Cells were then examined for ATP levels using CellTiter‐Glo (Promega) according to the manufacturer's instruction.

### Western blot and antibodies

Cell lysates were subject to SDS–PAGE on 4–20% Bis‐Tris gels and transferred to PVDF membranes. Pierce Fast Western Blot Kit (Thermo Scientific) was used to perform immunoblot according to the manufacturer's instructions with the following antibodies: mouse anti‐His‐tagged (Millipore 05‐949) and HRP‐conjugated mouse anti‐tubulin (Proteintech HRP‐66031). Chemiluminescence of anti‐His‐tagged antibody was performed with SuperSignal West Femto Maximum Sensitivity Substrate (Thermo Scientific).

### RNA‐seq analysis from SARS‐CoV‐2‐infected cells and COVID‐19‐infected patients

For the RNA‐seq data from SARS‐CoV‐2, IAV, HPIV3, and RSV infection (Blanco‐Melo *et al*, 2020), target gene expression in infected cells relative to uninfected controls were calculated and log2(fold change) was displayed as a heatmap. For single‐cell RNA sequencing analysis from COVID‐19‐infected patients, scRNA‐seq dataset for bronchoalveolar lavage fluid from four healthy controls, three moderate COVID‐19‐infected patients, and six severe COVID‐19‐infected patients were downloaded from GEO under the accession number GSE145926 (Liao *et al*, 2020). Analysis was based on the read count matrix, with cell type information provided by meta annotation text file. Reads counts of each gene were collected and calculated using the HDF5 matrix file provided. The read count box plot for HD, moderate, and severe COVID‐19‐infected patients are drawn based on this analysis.

### Statistical analysis

All data and graphs were analyzed and plotted using Prism 8.0.2 (GraphPad Inc.). Statistical analysis was performed using Student's *t*‐test (paired, two‐sided) if not specially stated in the figure legends. Data are presented as the means ± standard deviation (SD).

## Author contributions

SW designed and performed experiments, analyzed the data, and wrote the manuscript; WL performed experiments, analyzed the data, and wrote the manuscript; HH performed bioinformatics analyses, SKT designed and performed experiments and analyzed the data; QZ performed experiments and analyzed the data; SR and DS provided resources; BAC gave advice in experimental plans and performed experiments; AFC gave advice in experimental plans, performed experiments, and analyzed the data; TMR conceived the overall project and participated in experimental design, data analyses, interpretations, and manuscript writing.

## Conflict of interest

T.M.R. is a founder of ViRx Pharmaceuticals and has an equity interest in the company. The terms of this arrangement have been reviewed and approved by the University of California San Diego in accordance with its conflict of interest policies.

## Supporting information



Expanded View Figures PDF

Source Data for Figure 1

Source Data for Figure 3

Source Data for Figure 4

Source Data for Expanded View

Review Process File

## Data Availability

This study includes no data deposited in external repositories.
