# Supplementary material for: Cholesterol 25‐Hydroxylase inhibits SARS‐CoV‐2 and other coronaviruses by depleting membrane cholesterol
Source: EMBO J. 2020 Oct 5;39(21):e106057. doi: 10.15252/embj.2020106057 (PMC7537045; doi:10.15252/embj.2020106057)
Supplement: Supplementary file 1 — Expanded View Figures PDF [file EMBJ-39-e106057-s001.pdf]

Expanded View Figures

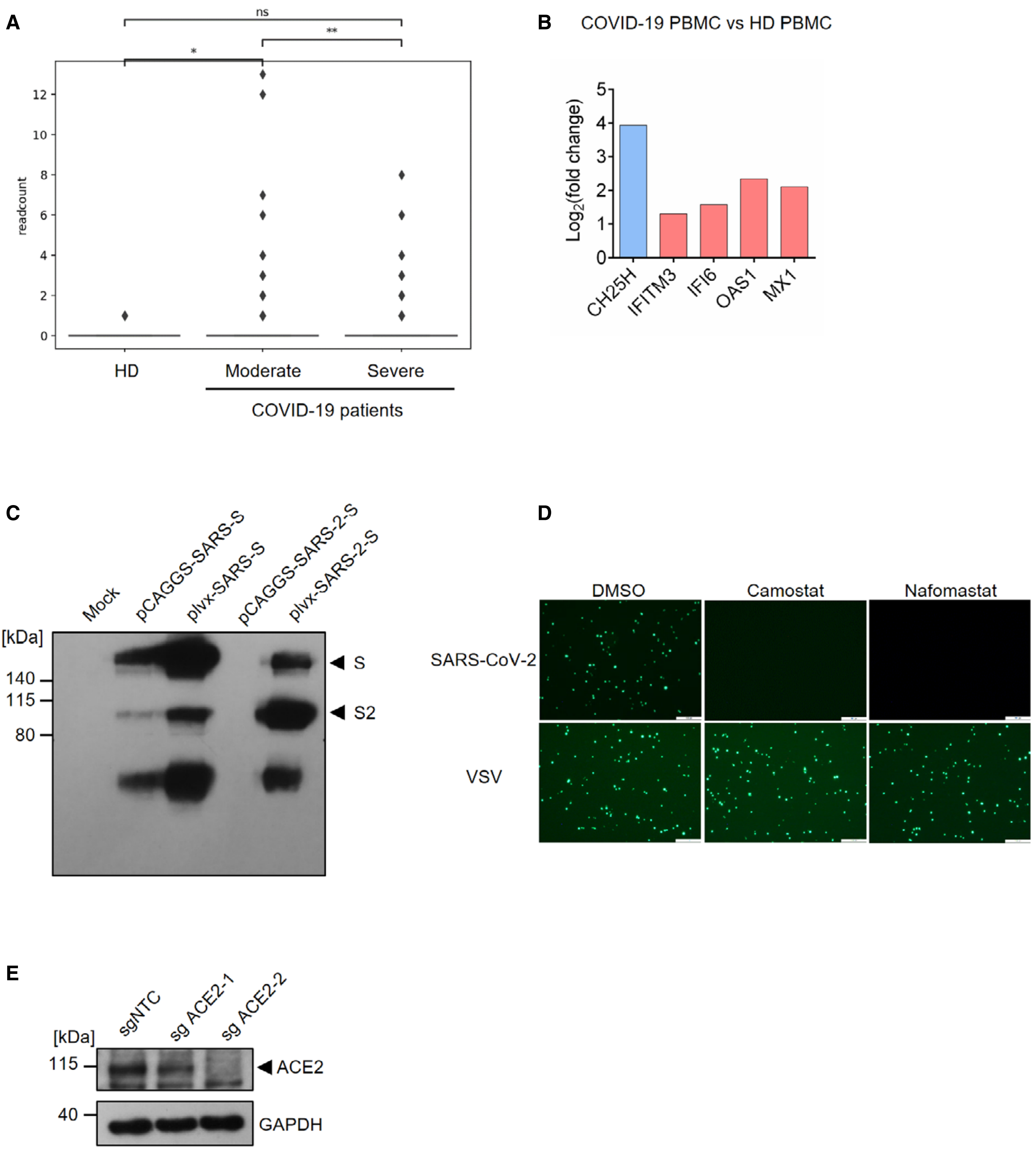

Figure EV1.

© 2020 The Authors

◀ **Figure EV1. (Related to Fig 1). *CH25H* induction in COVID-19-infected patient and characterization of SARS-CoV-2 pseudovirus.**

A The box plot shows the expression of *CH25H* in epithelia of bronchoalveolar lavage fluids from four healthy donors, three moderate COVID-19-infected patients and six severe COVID-19-infected patients by scRNA-seq analysis (Liao *et al*, 2020). *$P < 0.05$, **$P < 0.01$, by Student's *t*-test

B RNA-seq analysis showed robust induction of *CH25H* in PBMCs from COVID-19-infected patients relative to healthy donors (Blanco-Melo *et al*, 2020).

C Overexpression of SARS-CoV-2 and SARS-CoV spike protein in 293FT cells. The plasmids encoding SARS-CoV-2 or SARS-CoV spike protein was transfected in 293FT cells and expression of spike protein was analyzed by Western blotting using an antibody recognizing S2 subunit of SARS-CoV and SARS-CoV-2. Black arrows indicate full length spike protein and cleaved spike (S2 subunit), respectively.

D TMPRSS2 inhibitors, camostat and nafamostat, blocked entry of SARS-CoV-2 but not VSV. Calu-3 cells were treated with 10 μM camostat, 10 μM nafamostat or DMSO for 1 h. Then, the cells were infected with SARS-CoV-2 or VSV pseudovirus with EGFP for 1 h. EGFP signals were captured by fluorescence microscope 24 h post-infection. Scale bar, 100 μM.

E Knockout efficiency of ACE2 in Calu-3 cells. Calu-3 cells were transduced with non-targeting control or ACE2-specific sgRNA. ACE2 expression was analyzed by Western blotting. Black arrow indicates the band for ACE2.

Source data are available online for this figure.

© 2020 The Authors

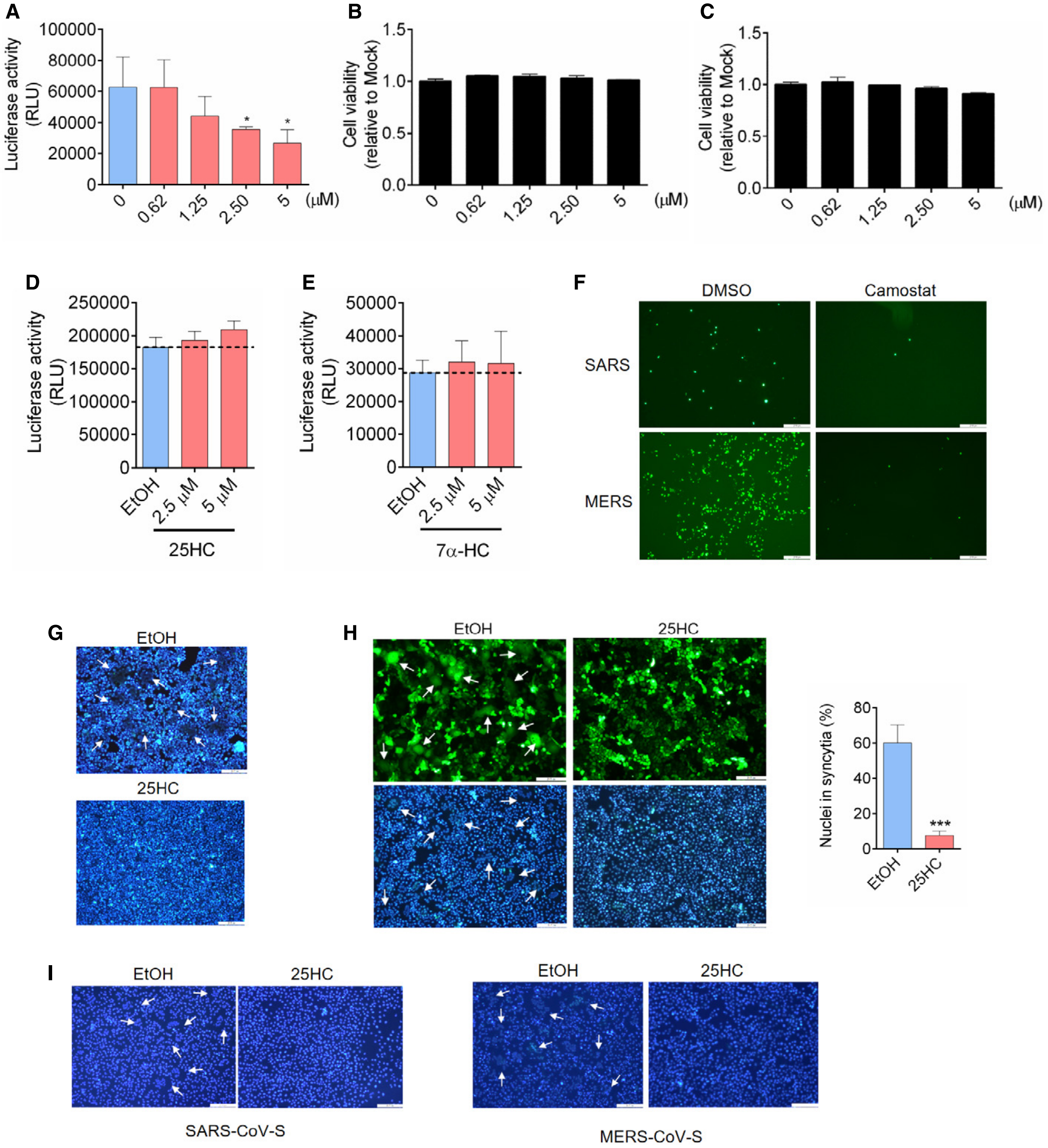
A
Luciferase activity (RLU)
100000
80000
60000
40000
20000
0
0
0.62
1.25
2.50
5
(μM)
B
Cell viability (relative to Mock)
1.5
1.0
0.5
0.0
C
D
250000
200000
150000
100000
50000
EtOH
2.5 μM
5 μM
25HC
E
50000
40000
30000
20000
10000
7α-HC
F
DMSO
Camostat
SARS
MERS
G
EtOH
25HC
H
Nuclei in syncytia (%)
80
60
40
20
***
I
SARS-CoV-S
MERS-CoV-S

Figure EV2.

Figure EV2. (Related to Fig 2). 25HC is not cytotoxic to Caco-2 and Calu-3 cells and inhibits coronavirus S-induced membrane fusion.

A 25HC inhibits SARS-CoV-2 pseudovirus entry into Caco-2 cells. Following a 16-h incubation with EtOH or indicated concentrations of 25HC, cells were challenged with SARS-CoV-2 pseudovirus encoding FLuc. After virus removal at 2 h, cells were lysed and luciferase activity was measured after 24 h. Statistical analyses were conducted by Student's *t*-test. Bar represents mean ± SD of $n = 3$. $^*P < 0.05$.

B, C 25HC is not cytotoxic to Caco-2 (B) or Calu-3 cells (C). Cell were treated with EtOH or indicated concentrations of 25HC for 24 h, and cell viability was measured by CellTiter-Glo (Promega). Bar represents mean ± SD of $n = 3$.

D 25HC does not affect VSV genome replication. Cell were infected with VSV pseudovirus encoding FLuc for 2 h. Then, medium with EtOH or 25HC was added. At 24 h post-infection, cells were lysed, and luciferase activity was measured. VSV replication was quantified by luciferase assays. Bar represents mean ± SD of $n = 3$.

E 7α-HC does not inhibit SARS-CoV-2 entry. Following a 16-h incubation with EtOH or indicated concentrations of 7α-HC, cells were challenged with SARS-CoV-2 pseudovirus encoding FLuc for 2 h. At 24 h post-infection, cells were lysed, and luciferase activity was measured. Statistical analyses were conducted by Student's *t*-test. Bar represents mean ± SD of $n = 3$. $^*P < 0.05$.

F TMPRSS2 inhibitors camostat blocked entry of SARS-CoV and MERS-CoV pseudoviruses. Calu-3 cells were treated with 10 μM camostat or DMSO for 1 h. Then the cells were infected with SARS-CoV or MERS-CoV pseudovirus with EGFP for 1 h. EGFP signals were captured by fluorescence microscope 24 h post-infection. Scale bar, 100 μm

G Hoechst stain of nuclei in Fig 2F. White arrows indicate syncytia. Scale bar, 50 μm.

H 25HC inhibits cell–cell fusion on Vero cells. Vero cells were treated with EtOH or 25HC (5 μM) for 16 h prior to transfection with pLVX plasmids encoding SARS-CoV-2 S. After 4 h, medium was changed and re-supplemented with EtOH or 25HC. At 48 h post-transfection, syncytium formation was visualized after trypsin treatment by fluorescence microscopy. White arrows indicate syncytia. Scale bar, 50 μm. Bar represents mean ± SD of $n = 3$. $^{***}P < 0.001$, by Student's *t*-test.

I Hoechst stain of nuclei in Fig 2G. White arrows indicate syncytia. Scale bar, 50 μm.

© 2020 The Authors

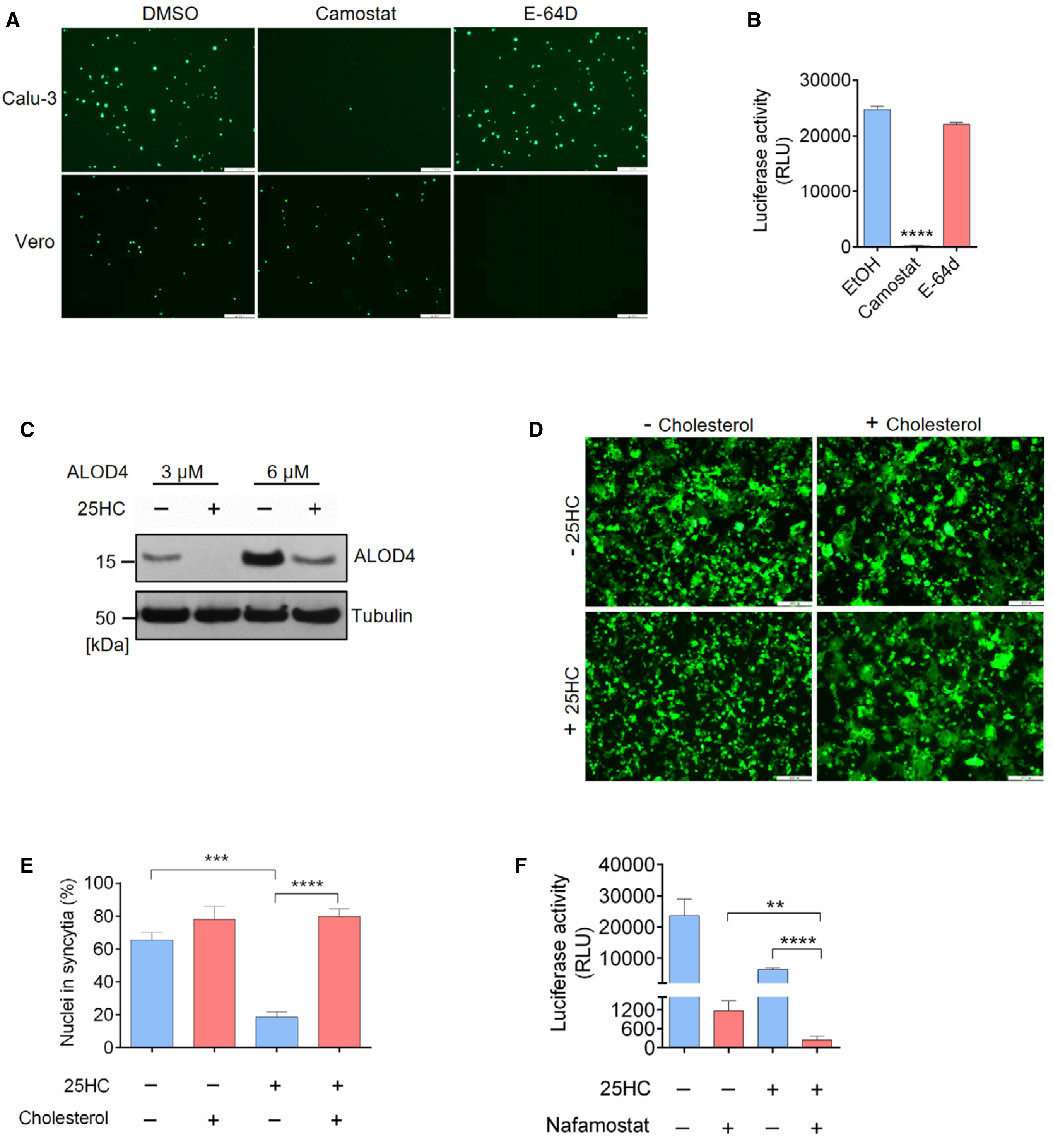

Figure EV3.

 © 2020 The Authors

Figure EV3. (related to Fig 3). Rescue of cholesterol depletion on the plasma membrane reverses SARS-CoV-2 spike-mediated cell–cell fusion.

A, B SARS-CoV-2 entry is dependent on TMPRSS2 but not endosomal cathepsin in lung epithelial cells. Calu-3 cells were treated with 10 μM camostat or 25 μM E-64D for 1 h. Then, the cells were infected with SARS-CoV-2 pseudovirus with EGFP (A) or Fluc (B) for 1 h. EGFP signals were captured by fluorescence microscope 24 h post-infection. Scale bar, 100 μm. Luciferase activity was measured at 24 h post-infection. Bar represents mean ± SD of $n = 3$. ****$P < 0.0001$, by Student's *t*-test.

C 25HC depletes accessible cholesterol on the plasma membrane. Calu-3 cells were treated with EtOH or 5 μM 25HC and subsequently incubated with 3 or 6 μM ALOD4 for 30 min. The amount of bound ALOD4 was examined by Western blotting.

D, E Supplement of cholesterol can rescue cell–cell fusion induced by SARS-CoV-2 S. (D) 293FT cells were treated with EtOH or 25HC (5 μM) for 16 h prior to transfection with pLVX plasmids encoding SARS-CoV-2 S. 4 h post-transfection, medium was changed and re-supplemented with EtOH or 25HC in the presence or absence of 80 μM cholesterol. 24 h post-transfection, syncytium formation was visualized by fluorescence microscopy. Scale bar, 50 μm. (E) Membrane fusion was quantified as the percentage of nuclei within syncytia out of all nuclei in GFP-positive cells. Bar represents mean ± SD of $n = 3$, ***$P < 0.001$, ****$P < 0.0001$, by Student's *t*-test.

F Synergistic effects of 25HC and TMPRSS2 inhibitor to block SARS-CoV-2 entry. Addition of Nafamostat in 25HC-treated cells were infected with SARS-CoV-2 pseudovirus and 24 h post-infection, cells were lysed, and luciferase activity was measured. Statistical analyses were conducted by Student's *t*-test. Bar represents mean ± SD of $n = 3$. **$P < 0.01$, ****$P < 0.0001$.

Source data are available online for this figure.

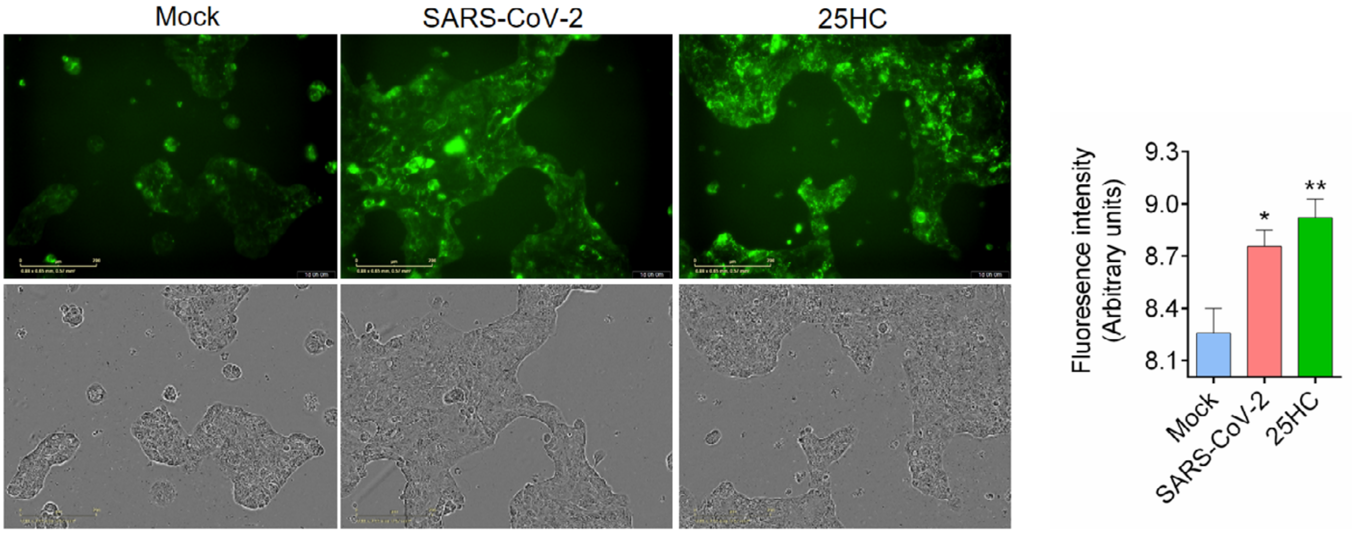

Figure EV4. (related to Fig 4). SARS-CoV-2 isolate USA-WA1/2020 activates ACAT.

Calu-3 cells were infected with SARS-CoV-2 USA-WA1/2020 at MOI = 2 for 24 h at 37°C or treated with 5 μM 25HC for 24 h. Cells were then stained with 1X LipidSpot 488 for 30 min. Fluorescence intensity was quantified by Incucyte S3 analysis software. Scale bar, 200 μm.

© 2020 The Authors
