# Supplementary figures and images for: Cholesterol 25‐Hydroxylase inhibits SARS‐CoV‐2 and other coronaviruses by depleting membrane cholesterol
Source: EMBO J. 2020 Oct 5;39(21):e106057. doi: 10.15252/embj.2020106057 (PMC7537045; doi:10.15252/embj.2020106057)

Figure 1C

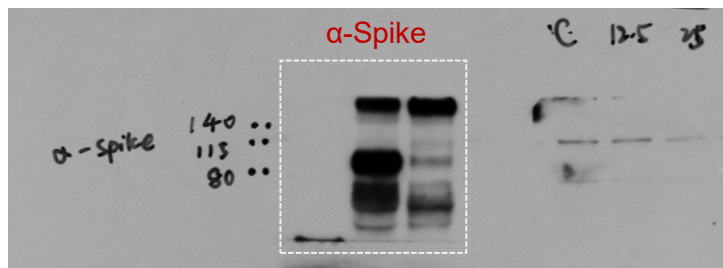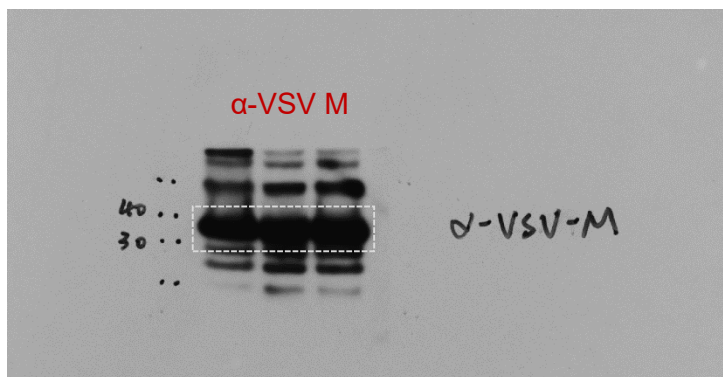

Supplement: Supplementary file 2 — Source Data for Figure 1 [file EMBJ-39-e106057-s002.pdf]

Figure 3B

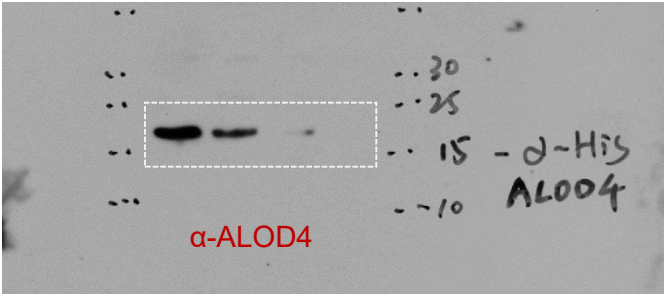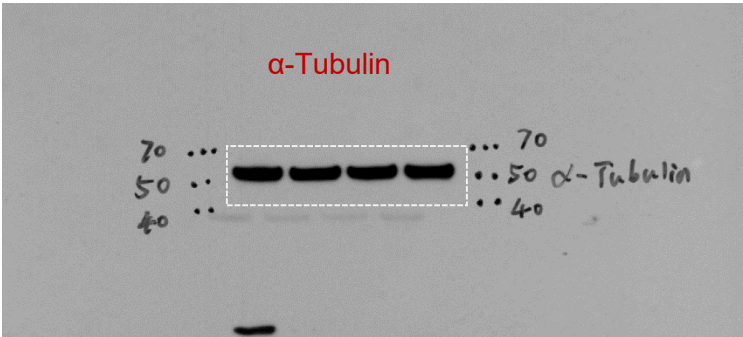

Figure 3D

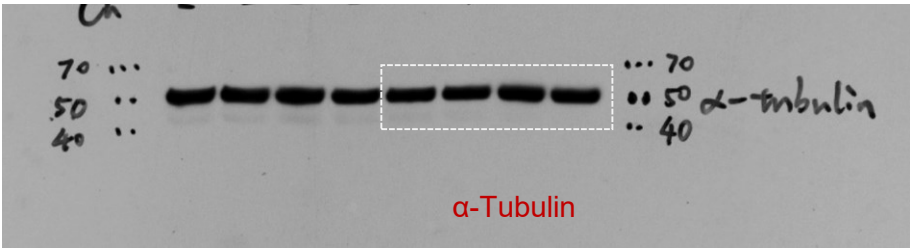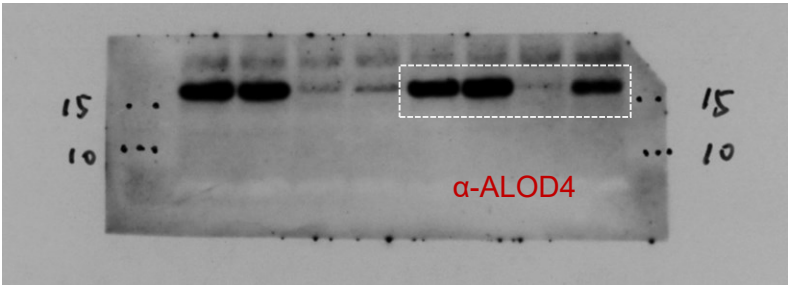

Supplement: Supplementary file 3 — Source Data for Figure 3 [file EMBJ-39-e106057-s003.pdf]

Figure 4C

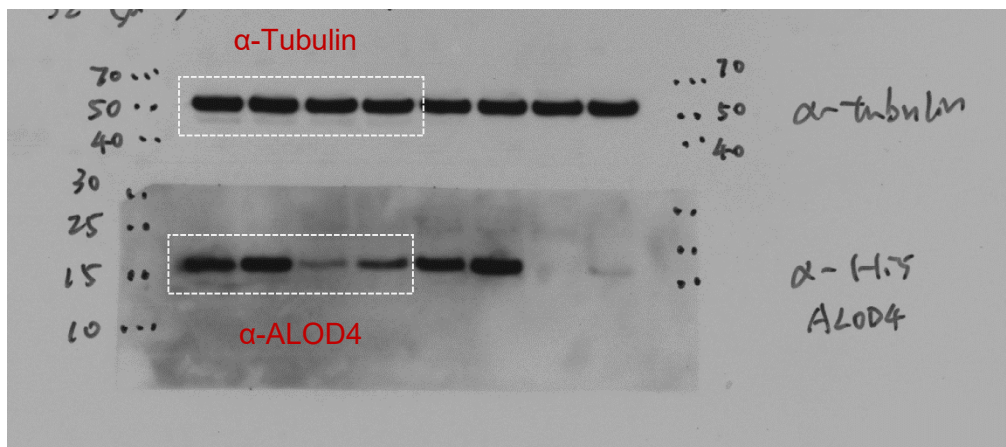

Supplement: Supplementary file 4 — Source Data for Figure 4 [file EMBJ-39-e106057-s004.pdf]

Figure EV3C

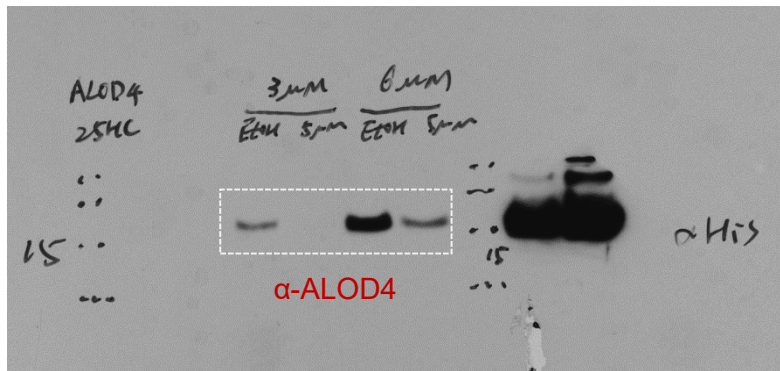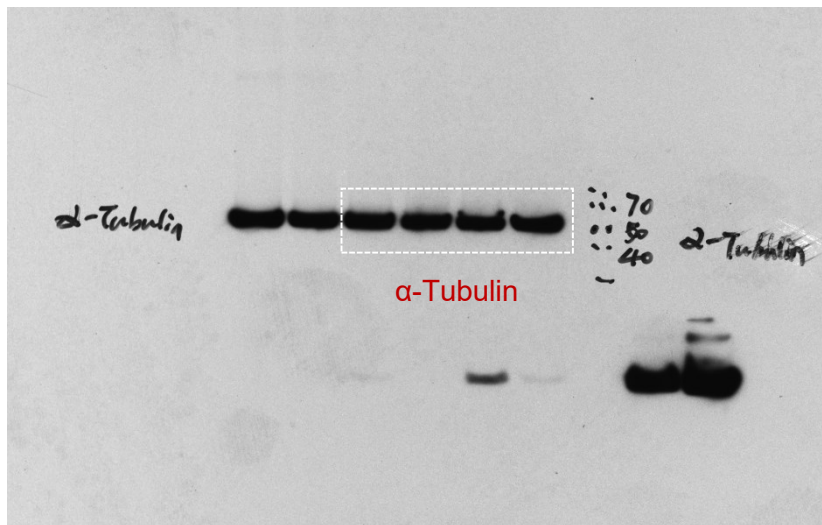

Supplement: Supplementary file 5 — Source Data for Expanded View [file EMBJ-39-e106057-s005.zip › EV_Figure_source_data/FigureEV3_Source_Data.pdf]

Figure EV1C

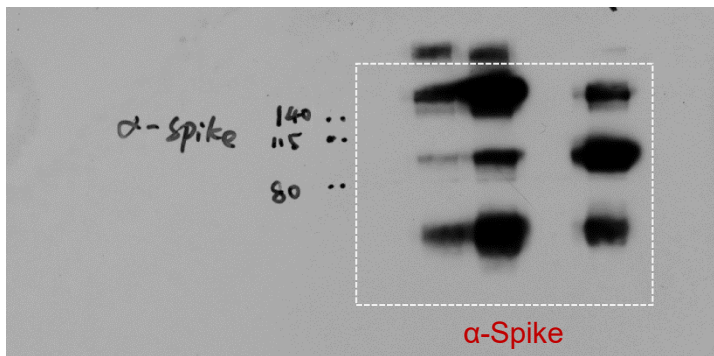

Figure EV1E

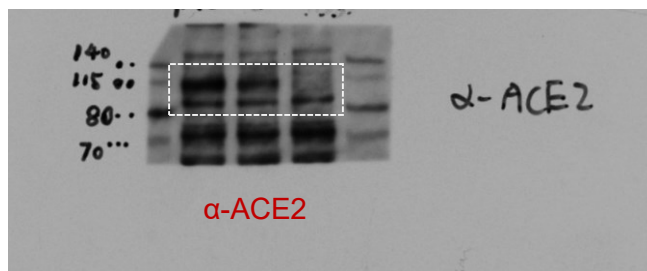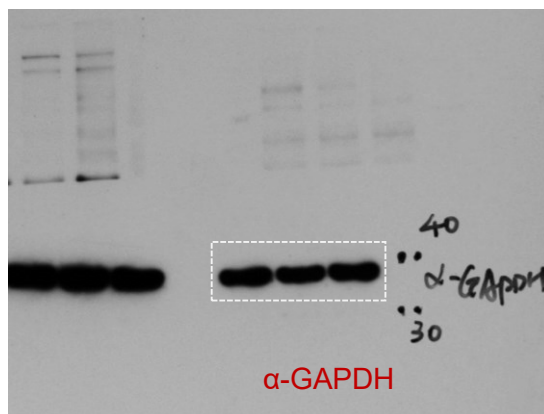

Supplement: Supplementary file 5 — Source Data for Expanded View [file EMBJ-39-e106057-s005.zip › EV_Figure_source_data/FigureEV1_Source_Data.pdf]
